# Mental Health Disparities by Sexual Orientation and Gender Identity in the All of Us Research Program

**DOI:** 10.1001/jamanetworkopen.2024.56264

**Published:** 2025-01-29

**Authors:** Junjie Anderson Lu, Shamsi Soltani, S. Bryn Austin, David H. Rehkopf, Mitchell R. Lunn, Marvin E. Langston

**Affiliations:** 1 Department of Epidemiology and Population Health, Stanford University School of Medicine, Stanford, California; 2 Department of Social and Behavioral Sciences, Harvard T. H. Chan School of Public Health, Boston, Massachusetts; 3 Division of Adolescent and Young Adult Medicine, Boston Children’s Hospital, Boston, Massachusetts; 4 Department of Pediatrics, Harvard Medical School, Boston, Massachusetts; 5 Division of Primary Care and Population Health, Department of Medicine, Stanford University School of Medicine, Stanford, California; 6 Department of Health Policy, Stanford University School of Medicine, Stanford, California; 7 Department of Sociology, Stanford University, Stanford, California; 8 Department of Pediatrics, Stanford University School of Medicine, Stanford, California; 9 Division of Nephrology, Department of Medicine, Stanford University School of Medicine, Stanford, California

## Abstract

**Importance:**

Limited research explores mental health disparities between individuals in sexual and gender minority (SGM) populations and cisgender heterosexual (non-SGM) populations using national-level data.

**Objective:**

To explore mental health disparities between SGM and non-SGM populations across sexual orientation, sex assigned at birth, and gender identity within the All of Us Research Program.

**Design, Setting, and Participants:**

This cross-sectional study used survey data and linked electronic health records of eligible All of Us Research Program participants from May 31, 2017, to June 30, 2022.

**Exposure:**

Self-identified SGM status.

**Main Outcomes and Measures:**

Prevalence of common mental health conditions identified from linked electronic health records. Logistic regression adjusted for age, race and ethnicity, educational level, income, employment status, and geographic region was used to assess the association between SGM status and mental health conditions.

**Results:**

Among 413 457 participants, 269 947 (65.3%) were included in the analysis (median age, 59 [IQR, 43-70] years), with 22 189 (8.2%) self-identified as SGM. Men with cisgender sexual minority identity had higher odds of bipolar disorder (adjusted odds ratio [AOR], 1.87; 95% CI, 1.70-2.56) compared with cisgender heterosexual men. Women with cisgender sexual minority identity had higher odds of bipolar disorder (AOR, 2.09; 95% CI, 1.95-2.25) compared with cisgender heterosexual women. Gender diverse people assigned female sex at birth had higher odds of posttraumatic stress disorder (PTSD) compared with both cisgender heterosexual men (AOR, 3.67; 95% CI, 2.99-4.50) and cisgender heterosexual women (AOR, 2.77; 95% CI, 2.26-3.40). Gender diverse individuals assigned male sex at birth had higher odds of bipolar disorder (AOR, 2.35; 95% CI, 1.66-3.33) compared with cisgender heterosexual men and higher odds of attention-deficit/hyperactivity disorder (AOR, 2.19; 95% CI, 1.48-3.23) compared with cisgender heterosexual women. Transgender men had higher odds of depression (AOR, 2.11; 95% CI, 1.80-2.49) compared with cisgender heterosexual men, while transgender women had higher odds of any personality disorder (AOR, 2.71; 95% CI, 1.84-3.99) compared with cisgender heterosexual women.

**Conclusions and Relevance:**

In this cross-sectional study of participants in the All of Us Research Program, there were significant mental health disparities between participants in SGM and non-SGM groups. These findings underscore the need for tailored mental health interventions to improve the well-being of SGM populations, while noting that the associations do not imply causality but reflect the stigma and minority stress experienced by these individuals.

## Key Points

**Question:**

Do the sexual and gender minority (SGM) populations have a higher prevalence of diagnosed mental health conditions compared with the cisgender and heterosexual (non-SGM) populations in the All of Us Research Program?

**Findings:**

In this cross-sectional study involving 413 457 participants, 269 947 were included in the analysis, of whom 22 189 self-identified as SGM. SGM subgroups had significantly higher odds of at least 4 of 10 commonly diagnosed mental health conditions compared with their non-SGM counterparts.

**Meaning:**

These findings underscore the need for systemic support in prevention and early intervention among SGM populations with mental health conditions.

## Teaser

This cross-sectional study uses data from the All of Us Research Program to investigate whether sexual and gender minority (SGM) populations have a higher prevalence of diagnosed mental health conditions compared with cisgender and heterosexual (non-SGM) populations.

## Introduction

Individuals in sexual and gender minority (SGM) groups—encompassing lesbian, gay, bisexual, transgender, queer, and additional noncisgender and/or nonheterosexual identities—routinely encounter societal stigma, discrimination, and minority stress.^1,2,3^ These societal challenges are associated with mental health disparities between SGM individuals and their cisgender heterosexual counterparts (non-SGM), with SGM populations exhibiting elevated incidences of depression, anxiety, and suicidality.^4,5,6,7,8,9,10,11,12,13,14^ For instance, the Gallup National Health and Well-Being Index and Behavioral Risk Factor Surveillance System found that SGM people are approximately 4.5 and 7.3 times as likely to be diagnosed with depression relative to non-SGM people.^15^ Similarly, a Medline literature review reported that sexual minority youths had 82% to 317% higher odds of experiencing depressive symptoms compared with their heterosexual peers.^16^

Sexual orientation, sex assigned at birth, and gender identity are distinct but interconnected domains.^17,18,19^ Considering these different domains is crucial to addressing the health needs of SGM populations.^20^ The lack of comprehensive datasets has historically limited the understanding of SGM health disparities, as traditional datasets often fail to analyze nuanced SGM subgroups.^21,22,23,24,25,26,27^ Moreover, the reliance on self-reported data for mental health condition assessments may lead to outcome misclassification, either due to recall bias or the social desirability bias, where individuals might underreport mental health diagnoses to avoid the stigma associated with these conditions.^28,29^

The All of Us Research Program, a national cohort study designed to engage 1 million participants from varied US demographic groups, offers a unique opportunity to explore mental health disparities.^30^ Detailed survey questions about sexual orientation and gender identity enable researchers to better understand subgroups within the SGM population.^8^ Additionally, the All of Us Research Program includes linkage to electronic health records (EHR), allowing researchers to obtain diagnosed mental health conditions of participants with good accuracy.^31,32^ EHR data capture clinically diagnosed conditions that may not be fully disclosed or accurately reported in self-administered surveys, providing a robust complement to survey data by reducing the risk of outcome misclassification.^33^

Minority stress theory highlights the impact of social stressors, including discrimination, on the mental health of SGM individuals, while ecosocial frameworks emphasize the interaction of social and environmental factors in shaping health outcomes.^34,35^ Drawing on these frameworks, this study aims to examine mental health conditions (eg, anxiety, depression, and posttraumatic stress disorder [PTSD]) within SGM subgroups using EHR-derived information and to identify the disparities between SGM and non-SGM populations. We hypothesized that SGM individuals would have a higher prevalence of these conditions compared with their non-SGM counterparts, and the prevalence would vary across the SGM subgroups. Understanding mental health disparities is essential for designing interventions that reduce health disparities and promote overall well-being in SGM communities.

## Methods

### Data Sources and Population

This study aimed to examine mental health disparities among participants within the All of Us Research Program. Detailed study methodology for All of Us is described elsewhere.^30^ For the present study, we performed a cross-sectional analysis using the Controlled Tier Data, version 7. Participants’ self-reported SGM status and the prevalence of diagnosed mental health outcomes were measured by “the Basics” participant-provided information module (survey) and linked EHR, respectively. The All of Us Research Program Institutional Review Board approved all procedures, with participants providing consent to share their health data. The Institutional Review Board determined that analyses using Controlled Tier Data within the Researcher Workbench do not qualify as human participants research. We followed the Strengthening the Reporting of Observational Studies in Epidemiology (STROBE) guideline for cross-sectional studies.

**Figure 1.**
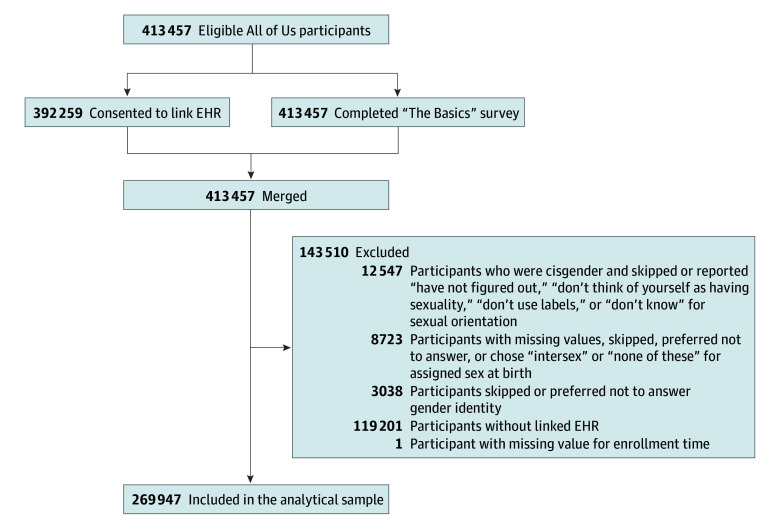
Flowchart for Participants in the All of Us Research Program (2017-2022) EHR indicates electronic health record.

Figure 1 shows the participant flowchart. From the initial of 413 457 All of Us participants enrolled from May 31, 2017, to June 30, 2022, we excluded the following participants: (1) cisgender participants who did not answer or selected “have not figured out,” “don’t think of yourself as having sexuality,” “don’t use labels,” or “don’t know” for sexual orientation (n = 12 547); (2) participants with missing values, who identified as “intersex,” who selected “none of these,” did not answer, or who selected “prefer not to disclose” their sex assigned at birth (n = 8723); (3) participants who skipped the question or selected “prefer not to answer” for gender identity (n = 3038); (4) participants who did not have a linked EHR (n = 119 201); and (5) 1 participant who had a missing value for the date of enrollment.

### Measurements

#### SGM Status

We referenced the demonstration project by Tran et al^8^ to define 8 mutually exclusive groups based on participants’ sex assigned at birth, sexual orientation identity, and gender identity (eTable 2 in Supplement 1): (1) cisgender heterosexual men; (2) cisgender heterosexual women; (3) cisgender sexual minority men; (4) cisgender sexual minority women; (5) gender diverse (ie, outside binary man or woman) people of any sexual orientation assigned female sex at birth; (6) gender diverse people of any sexual orientation assigned male sex at birth; (7) transgender men of any sexual orientation, and (8) transgender women of any sexual orientation. Specifically, cisgender participants with sexual minority identity included people who did not select straight as their only sexual orientation and chose other sexual orientation options.

#### Mental Health Conditions

We selected 10 common mental health conditions in the US as outcomes of interest, based on current prevalence and treatment statistics from the National Institute of Mental Health. These included (1) anxiety, (2) attention-deficit/hyperactivity disorder (ADHD), (3) autism spectrum disorder (ASD), (4) bipolar disorder (I or II), (5) eating disorder, (6) depression, (7) obsessive-compulsive disorder (OCD), (8) any personality disorder, (9) PTSD, and (10) schizophrenia.^36^

The All of Us Research Program uses the Observational Medical Outcomes Partnership data model to standardize EHR data. Systematized Nomenclature of Medicine (SNOMED) codes were queried from this harmonized dataset (eTable 1 in Supplement 1) to identify diagnosed mental health conditions. To decrease the possible outcome misclassifications, we defined the mental health condition as having the SNOMED code on at least 2 separate dates for the participant during the study period.^8,31^

#### Covariates

The selection of covariates was guided by the ecosocial theory, minority stress theory, and the structural definition of confounding. Ecosocial theory emphasizes the role of social and environmental factors in shaping health outcomes, while minority stress theory highlights the impact of chronic social stressors on the mental health of marginalized groups.^37,38,39^ The covariates were assumed to be associated with both the measurements of SGM status and the mental health outcomes,^40,41^ including the current age (continuous), annual household income (categorical), employment for wages (categorical), study enrollment years (categorical), US census division (categorical), and ethnic and racial identity (categorical). Specifically, income, employment status, and race and ethnicity are social determinants that can vary by SGM status due to discrimination and resource access, while enrollment year and US census division account for temporal and regional differences in SGM identification and mental health diagnoses. Race and ethnicity categories were self-reported and included African American or Black, Asian, Hispanic or Latinx, Middle Eastern or North African, Native Hawaiian or Other Pacific Islander, and White. Categories were not mutually exclusive and participants could pick as many as applied. Race and ethnicity data were collected because they were controlled for as confounding factors and social determinants of health.

### Statistical Analysis

Participants’ sociodemographic characteristics were presented by SGM and non-SGM subgroups after applying exclusion criteria. We used a complete case approach; participants without linked EHR data or with any missing covariate value were excluded from the analysis (Figure 1). The proportion of participants without a linked EHR in both SGM and non-SGM subgroups was assessed to determine whether there were differences in the extent of missing data between these groups (eFigure 1 in Supplement 1). We found no systematic sociodemographic differences between participants included and excluded from the analytical sample, as the standardized mean differences for the variables between these 2 groups were less than 0.2 (eTable 3 in Supplement 1).^42^

In the primary analysis, we examined the association between specific SGM subgroups and diagnosed mental health conditions by comparing each SGM subgroup with an appropriate cisgender heterosexual reference group. We analyzed gender diverse individuals against both cisgender heterosexual men and women to explore the association of cisgender heterosexuality with mental health disparities following the demonstration project by Tran et al.^8^ We also assessed the association by SGM subgroup and the number of diagnosed mental health conditions using negative binomial regression models, controlling for the same covariates.

We conducted sensitivity analyses. Inverse probability weighting was used to control confounding, calculating weights using the same covariates as in the primary analysis. The standardized mean differences before and after adjustment were analyzed using Love plots, and the conditional exchangeability was assessed through the distribution of propensity scores (eFigures 5-7 in Supplement 1). To assess potential model misspecification by collinearity among the independent variables (eFigure 4 in Supplement 1), we removed the indicator variables for race and ethnicity. To examine potential outcome misclassifications, we introduced 2 alternative definitions for outcomes. First, we refined the primary outcome definition by requiring that participants not only have the diagnosis code recorded on at least 2 separate dates but also have at least 1 US Food and Drug Administration–approved prescription record for the specific mental health condition (eTable 13 in Supplement 1). Second, we refined the primary outcome definition by requiring that participants not only have the SNOMED code recorded on at least 2 separate dates but also that the code appear in both an inpatient and an outpatient visit (eTable 17 in Supplement 1). As an exploratory analysis, we report on the demographic characteristics and prevalence of diagnosed mental health conditions for intersex participants in eTables 21 and 22 in Supplement 1. The analyses were conducted in the All of Us Researcher Workbench in R, version 4.2 (R Project for Statistical Computing). Statistical significance was defined as a 95% CI excluding 1 and a 2-sided *P* < .05. In compliance with the All of Us data and statistics dissemination policy, we did not disclose cell counts ranging between 1 and 19. The data are publicly available to authorized users through the All of Us Researcher Workbench.

## Results

Among the 413 457 All of Us participants, 269 947 (65.3%) were included in the analysis, with a median age of 59 (IQR, 43-70) years, and 22 189 (8.2%) self-identified as SGM (Figure 1 and eTable 3 in Supplement 1). A total of 56 126 participants (20.8%) self-identified as African American or Black; 9587 (3.6%) as Asian; 50 836 (18.8%) as Hispanic or Latinx; 2751 (1.0%) as Middle Eastern or North African; 677 (0.3%) as Native Hawaiian or Other Pacific Islander; and 154 260 (57.1%) as White. Among SGM participants, most were cisgender sexual minority women (56.6%; 95% CI, 56.4%-56.8%) (Table 1). The median ages differ across subgroups (Kruskal-Wallis test, H_7_  statistic, 10 472; *P* < .001), though there was overlap in IQRs (eFigure 2 in Supplement 1). Income distributions also vary across these groups (χ^2^_35_ = 2712.8; *P <* .001) (eFigure 2 in Supplement 1). Post hoc pairwise comparisons are provided in eFigures 2 and 3 in Supplement 1. In the unadjusted analysis, SGM participants had higher odds of ADHD, ASD, bipolar disorder, personality disorder, and PTSD compared with non-SGM participants. For other mental health conditions, including anxiety, depression, and eating disorders, prevalence varied between SGM and non-SGM groups as detailed in Table 2 and eTables 4 to 11 in Supplement 1.

**Table 1.** Baseline Sociodemographic Characteristics of Participants by Sexual Orientation and Gender Identity Groups: All of Us Research Program (2017-2022)^a^

Characteristic	Participants, No. (%)^b^							
	Cisgender				Gender diverse of any sexual orientation		Transgender of any sexual orientation	
	Heterosexual men (n = 94 998)	Heterosexual women (n = 152 760)	Sexual minority men (n = 8075)	Sexual minority women (n = 11 572)	Assigned female sex at birth (n = 884)	Assigned male sex at birth (n = 365)	Men (n = 638)	Women (n = 655)
Age, median (IQR), y	61 (46-72)	57 (41-69)	54 (38-65)	40 (31-55)	33 (27-43)	37 (29-52)	43 (32-58)	49 (37-63)
Race and ethnicity^c^								
African American or Black	20 848 (21.9)	30 600 (20.0)	1728 (21.4)	2512 (21.7)	84 (9.5)	40 (11.0)	143 (22.4)	171 (26.1)
Asian	3253 (3.4)	5479 (3.6)	299 (3.7)	440 (3.8)	53 (6.0)	23 (6.3)	24 (3.8)	<20
Hispanic or Latinx	14 904 (15.7)	32 112 (21.0)	1320 (16.3)	1943 (16.8)	108 (12.2)	45 (12.3)	226 (35.4)	178 (27.2)
Middle Eastern or North African	1068 (1.1)	1428 (0.9)	93 (1.2)	118 (1.0)	20 (2.3)	<20	<20	<20
Native Hawaiian or Other Pacific Islander	241 (0.3)	353 (0.2)	23 (0.3)	48 (0.4)	<20	<20	<20	<20
White	55 246 (58.2)	85 451 (55.9)	4863 (60.2)	7202 (62.2)	691 (78.2)	260 (71.2)	252 (39.5)	295 (45.0)
Sexual orientation								
Asexual	0	0	62 (0.8)	209 (1.8)	64 (7.2)	<20	<20	<20
Bisexual	0	0	2115 (26.2)	6858 (59.3)	277 (31.3)	88 (24.1)	86 (13.5)	106 (16.2)
Gay	0	0	5535 (68.5)	398 (3.4)	36 (4.1)	83 (22.7)	47 (7.4)	57 (8.7)
Lesbian	0	0	30 (0.4)	3119 (27.0)	138 (15.6)	<20	38 (6.0)	66 (10.1)
Mostly straight	0	0	109 (1.3)	352 (3.0)	<20	<20	<20	<20
Queer	0	0	22 (0.3)	216 (1.9)	145 (16.4)	33 (9.0)	24 (3.8)	<20
Polysexual, omnisexual, sapiosexual, or pansexual	0	0	57 (0.7)	278 (2.4)	99 (11.2)	23 (6.3)	22 (3.4)	20 (3.1)
Straight	94 998 (100)	152 760 (100)	157 (1.9)	488 (4.2)	73 (8.3)	78 (21.4)	335 (52.5)	286 (43.7)
Two-Spirit	0	0	27 (0.3)	14 (0.1)	<20	<20	<20	<20
Gender identity								
Genderfluid, genderqueer, gender variant, unsure, specific gender, or two-spirit	0	0	0	0	108 (12.2)	72 (19.7)	0	0
Man	94 998 (100)	0	8075 (100)	0	0	0	359 (56.3)	0
Nonbinary	0	0	0	0	757 (85.6)	281 (77.0)	0	0
Transgender^d^	0	0	0	0	108 (12.2)	43 (11.8)	221 (34.6)	312 (47.6)
Woman	0	152 760 (100)	0	11 572 (100)	0	0	0	310 (47.3)
Annual household income, $								
≤24 999	24 892 (26.2)	37 130 (24.3)	2662 (33.0)	3943 (34.1)	308 (34.8)	138 (37.8)	279 (43.7)	285 (43.5)
25 000-49 999	12 532 (13.2)	24 239 (15.9)	1353 (16.8)	2170 (18.8)	187 (21.2)	71 (19.5)	83 (13.0)	101 (15.4)
50 000-99 999	17 032 (17.9)	29 193 (19.1)	1492 (18.5)	2086 (18.0)	156 (17.6)	53 (14.5)	68 (10.7)	61 (9.3)
100 000-149 999	9948 (10.5)	14 830 (9.7)	755 (9.3)	966 (8.3)	74 (8.4)	39 (10.7)	22 (3.4)	30 (4.6)
≥150 000	12 092 (12.7)	15 489 (10.1)	864 (10.7)	908 (7.8)	63 (7.1)	27 (7.4)	25 (3.9)	23 (3.5)
Prefer to not answer or skipped	18 502 (19.5)	31 879 (20.9)	949 (11.8)	1499 (13.0)	96 (10.9)	37 (10.1)	161 (25.2)	155 (23.7)
Some college or higher	62 383 (65.7)	108 487 (71.0)	6066 (75.1)	8197 (70.8)	749 (84.7)	285 (78.1)	310 (48.6)	323 (49.3)
Employed for wages	32 915 (34.6)	65 823 (43.1)	3436 (42.6)	5711 (49.4)	494 (55.9)	171 (46.8)	236 (37.0)	212 (32.4)
Own a home	44 966 (47.3)	73 626 (48.2)	2685 (33.3)	3381 (29.2)	204 (23.1)	81 (22.2)	134 (21.0)	152 (23.2)
Health insurance	85 575 (90.1)	142 734 (93.4)	7377 (91.4)	10 663 (92.1)	837 (94.7)	338 (92.6)	534 (83.7)	556 (84.9)
Enrollment year								
2017	2562 (2.7)	4849 (3.2)	245 (3.0)	355 (3.1)	24 (2.7)	<20	<20	<20
2018	22 895 (24.1)	39 037 (25.6)	2074 (25.7)	2765 (23.9)	168 (19.0)	61 (16.7)	154 (24.1)	164 (25.0)
2019	40 272 (42.4)	63 760 (41.7)	3278 (40.6)	4401 (38.0)	252 (28.5)	126 (34.5)	294 (46.1)	279 (42.6)
2020	10 856 (11.4)	16 343 (10.7)	847 (10.5)	1236 (10.7)	105 (11.9)	37 (10.1)	60 (9.4)	67 (10.2)
2021	10 459 (11.0)	16 585 (10.9)	1000 (12.4)	1635 (14.1)	201 (22.7)	80 (21.9)	62 (9.7)	79 (12.1)
2022	7954 (8.4)	12 186 (8.0)	631 (7.8)	1180 (10.2)	134 (15.2)	54 (14.8)	55 (8.6)	55 (8.4)

^a^ Descriptions of survey questions and possible answers are given in Supplement 1.

^b^ Sexual and gender minority (SGM) and non-SGM groups defined based on sex assigned at birth, sexual orientation, and gender identity. Cisgender sexual minority participants were those who did not select straight as their only sexual orientation and chose other sexual orientation options (eTable 2 in Supplement 1).

^c^ Categories do not sum to the column total because participants may self-identify in multiple groups.

^d^ Participants in gender diverse columns listed as transgender identified as both transgender and another gender minority identity, unlike those in transgender men and women columns, who solely identified as transgender and/or man or woman.

**Table 2.** Electronic Health Record–Diagnosed Mental Health Conditions of Participants by Sexual Orientation and Gender Identity Group in the All of Us Research Program (2017-2022)

Condition	Participants, No. (%) (N = 269 947)^a^							
	Cisgender				Gender diverse of any sexual orientation		Transgender of any sexual orientation	
	Heterosexual men (n = 94 998)	Heterosexual women (n = 152 760)	Sexual minority men (n = 8075)	Sexual minority women (n = 11 572)	Assigned female sex at birth (n = 884)	Assigned male sex at birth (n = 365)	Men (n = 638)	Women (n = 655)
Anxiety	15 039 (15.8)	36 593 (24.0)	1960 (24.3)	3583 (31.0)	319 (36.1)	104 (28.5)	179 (28.1)	160 (24.4)
ADHD	1804 (1.9)	2657 (1.7)	288 (3.6)	548 (4.7)	80 (9.0)	28 (7.7)	33 (5.2)	<20
ASD	177 (0.2)	77 (0.1)	35 (0.4)	52 (0.4)	20 (2.3)	<20	<20	<20
Bipolar disorder	3175 (3.3)	5368 (3.5)	550 (6.8)	1093 (9.4)	87 (9.8)	37 (10.1)	56 (8.8)	45 (6.9)
Eating disorder	238 (0.3)	1239 (0.8)	34 (0.4)	170 (1.5)	<20	<20	<20	<20
Depression	15 154 (16.0)	35 563 (23.3)	2139 (26.5)	3463 (29.9)	306 (34.6)	96 (26.3)	188 (29.5)	177 (27.0)
OCD	307 (0.3)	621 (0.4)	50 (0.6)	103 (0.9)	<20	<20	<20	<20
Personality disorder	1131 (1.2)	1789 (1.2)	168 (2.1)	360 (3.1)	42 (4.8)	<20	33 (5.2)	28 (4.3)
PTSD	3227 (3.4)	4806 (3.1)	369 (4.6)	936 (8.1)	113 (12.8)	31 (8.5)	51 (8.0)	52 (7.9)
Schizophrenia	1367 (1.4)	920 (0.6)	148 (1.8)	145 (1.3)	<20	<20	<20	22 (3.4)

Abbreviations: ADHD, attention-deficit/hyperactivity disorder; ASD, autism spectrum disorder; OCD, obsessive-compulsive disorder; PTSD, posttraumatic stress disorder.

^a^ Groups with 1 to 19 participants were described as having fewer than 20 in accordance with All of Us policy.

**Figure 2.**
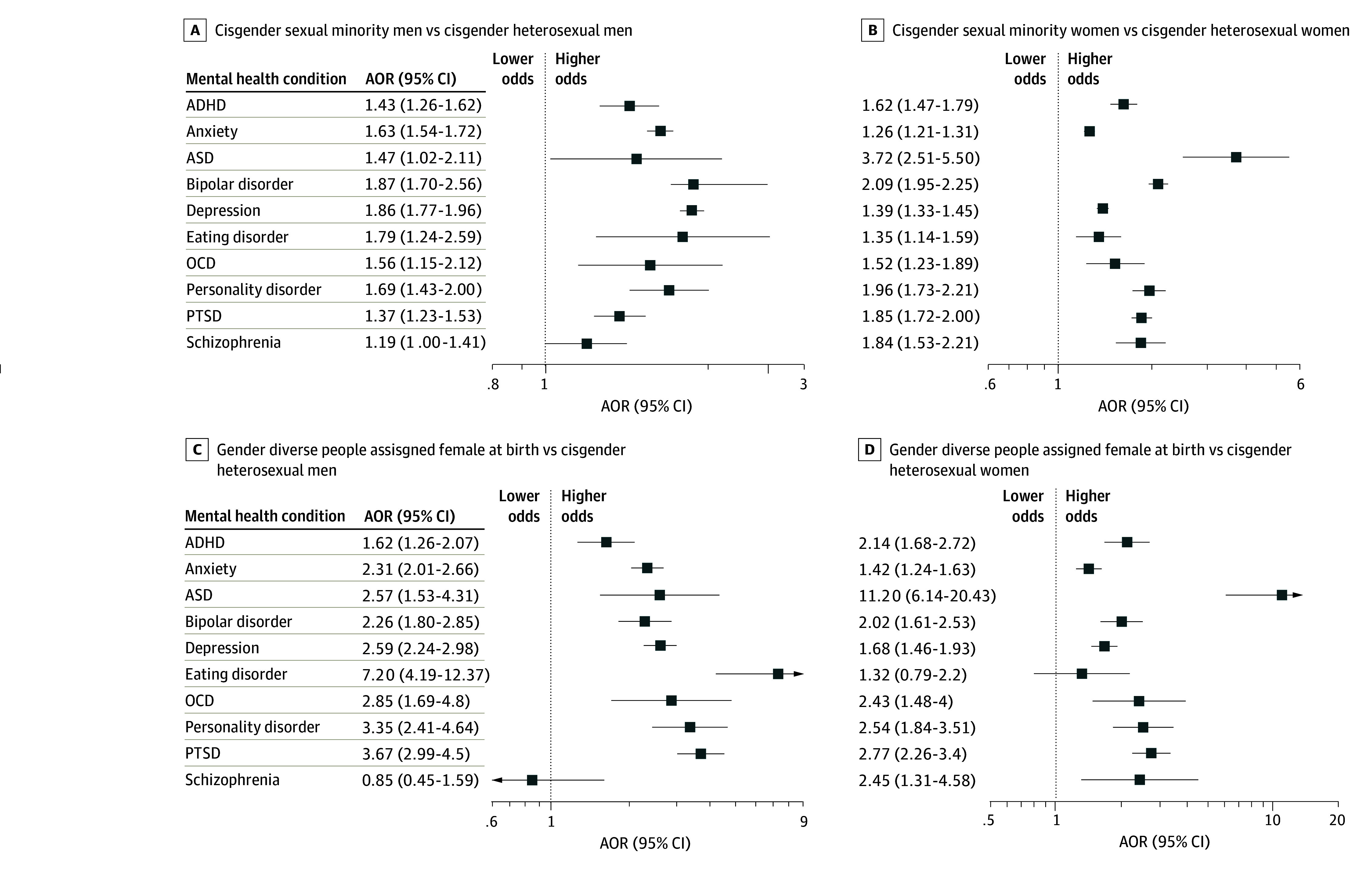
Adjusted Odds Ratios (AORs) of Diagnosed Mental Health Conditions Among Cisgender Sexual Minority Men, Cisgender Sexual Minority Women, and Gender Diverse People of Any Sexual Orientation Assigned Female Sex at Birth in the All of Us Research Program (2017- 2022) ADHD indicates attention-deficit/hyperactivity disorder; ASD, autism spectrum disorder; OCD, obsessive-compulsive disorder; and PTSD, posttraumatic stress disorder. Models were adjusted for current age, annual income, employment, enrollment year, US census division, and race and ethnicity.

Adjusted analyses showed that cisgender sexual minority men had higher odds of 9 of 10 commonly diagnosed mental health conditions compared with cisgender heterosexual men (eg, adjusted odds ratio [AOR] for bipolar disorder, 1.87; 95% CI, 1.70-2.56), except for schizophrenia (AOR, 1.19; 95% CI, 1.00-1.41) (Figure 2A and eTable 4 in Supplement 1). In contrast, cisgender sexual minority women had higher odds of all 10 mental health conditions (eg, AOR for bipolar disorder, 2.09; 95% CI, 1.95-2.25) compared with cisgender heterosexual women (Figure 2B and eTable 5 in Supplement 1).

**Figure 3.**
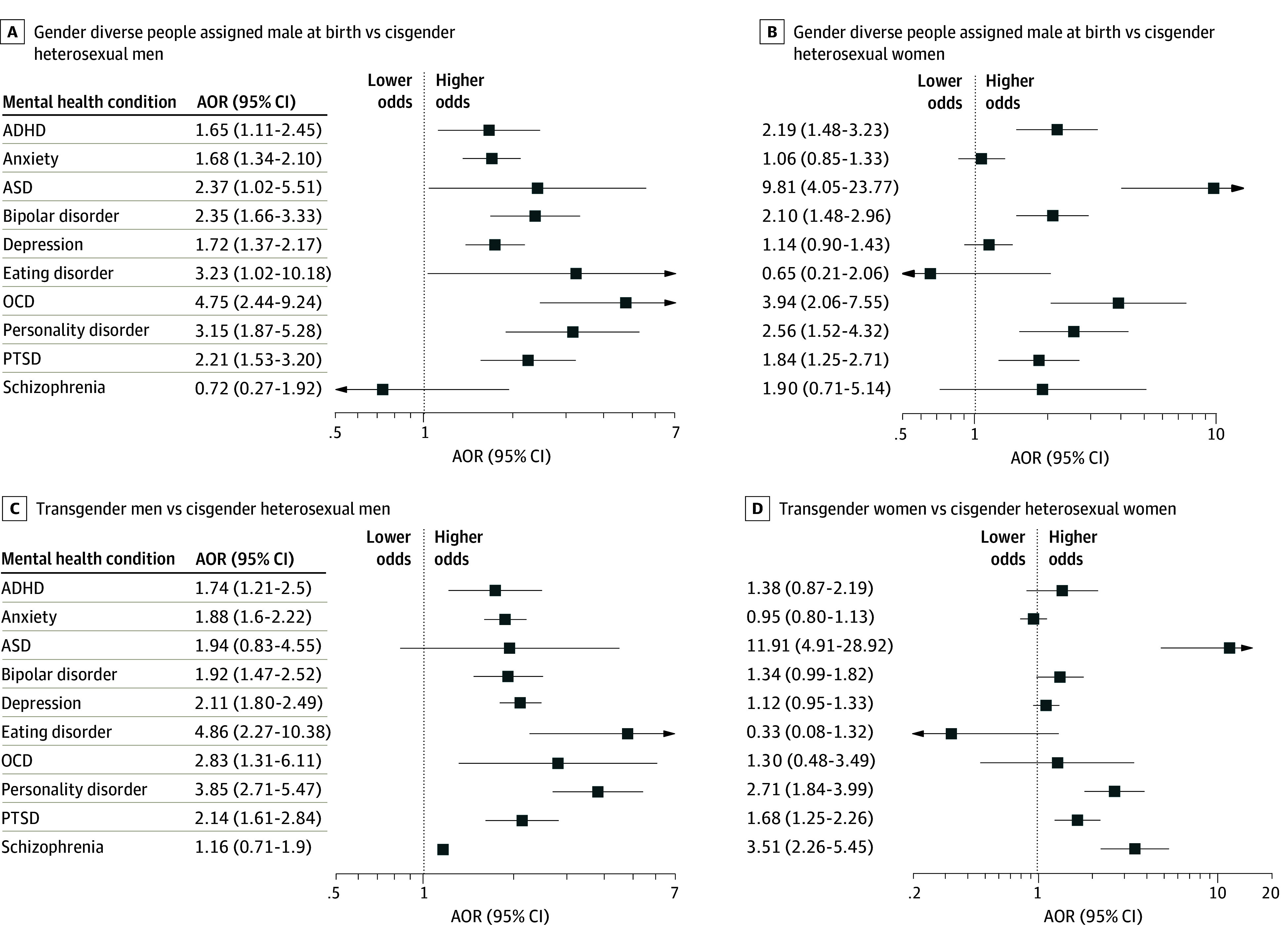
Adjusted Odds Ratios (AORs) of Diagnosed Mental Health Conditions Among Gender Diverse People of Any Sexual Orientation Assigned Male Sex at Birth, Transgender Men of Any Sexual Orientation, and Transgender Women of Any Sexual Orientation in the All of Us Research Program (2017- 2022) ADHD indicates attention-deficit/hyperactivity disorder; ASD, autism spectrum disorder; OCD, obsessive-compulsive disorder; and PTSD, posttraumatic stress disorder. Models were adjusted for current age, annual income, employment, enrollment year, US census division, and race and ethnicity.

Gender diverse people of any sexual orientation and assigned female sex at birth exhibited higher odds of 9 of 10 commonly diagnosed mental health conditions compared with cisgender heterosexual men, including PTSD (AOR, 3.67; 95% CI, 2.99-4.50), anxiety (AOR, 2.31; 95% CI, 2.01-2.66), and depression (AOR, 2.59; 95% CI, 2.24-2.98); the exception was for schizophrenia (AOR, 0.85; 95% CI, 0.45-1.59). Similarly, compared with cisgender heterosexual women, they had higher odds for 9 of 10 conditions (eg, AOR for PTSD, 2.77; 95% CI, 2.26-3.40), with the exception of eating disorders (AOR, 1.32; 95% CI, 0.79-2.20) (Figure 2C and D and eTables 6 and 7 in Supplement 1). Gender diverse people of any sexual orientation and assigned male sex at birth also had higher odds for 9 of 10 mental health conditions compared with cisgender heterosexual men (eg, AOR for bipolar disorder, 2.35; 95% CI, 1.66-3.33), except for schizophrenia (AOR, 0.72; 95% CI, 0.27-1.92). When compared with cisgender heterosexual women, they exhibited higher odds for ADHD (AOR, 2.19; 95% CI, 1.48-3.23), ASD (AOR, 9.81; 95% CI, 4.05-23.77), bipolar disorder (AOR, 2.10; 95% CI, 1.48-2.96), OCD (AOR, 3.94; 95% CI, 2.06-7.55), personality disorder (AOR, 2.56; 95% CI, 1.52-4.32), and PTSD (AOR, 1.84; 95% CI, 1.25-2.71) (Figure 3A and B and eTables 8 and 9 in Supplement 1).

Transgender men of any sexual orientation had higher odds for 8 of 10 mental health conditions compared with cisgender heterosexual men (eg, AOR for depression, 2.11; 95% CI, 1.80-2.49), except for ASD (AOR, 1.94; 95% CI, 0.83-4.55) and schizophrenia (AOR, 1.16; 95% CI, 0.71-1.90) (Figure 3C and eTable 10 in Supplement 1). Transgender women of any sexual orientation had higher odds of ASD (AOR, 11.91; 95% CI, 4.91-28.92), personality disorder (AOR, 2.71; 95% CI, 1.84-3.99), PTSD (AOR, 1.68; 95% CI, 1.25-2.26), and schizophrenia (AOR, 3.51; 95% CI, 2.26-5.45) compared with cisgender heterosexual women (Figure 3D and eTable 11 in Supplement 1). Sensitivity analyses yielded similar results (eTables 14-16 and 18-20 and eFigures 9-12 in Supplement 1). Compared with their cisgender heterosexual counterparts, SGM groups exhibited a higher rate of multiple concurrent mental health conditions, as depicted in eTable 12 and eFigure 8 in Supplement 1.

## Discussion

Our cross-sectional analysis of the All of Us Research Program data leveraged detailed survey questions about sexual orientation and gender identity to explore mental health disparities between individuals with SGM status and their non-SGM counterparts. We observed that the SGM groups exhibited higher odds of at least 4 of 10 diagnosed mental health conditions compared with their cisgender heterosexual counterparts. This pattern persisted after conducting multiple sensitivity analyses.

To our knowledge, this represents the most extensive collection of SGM and non-SGM participants within a national cohort, complete with EHR linkage. Health care barriers faced by SGM individuals can lead to underdiagnosed mental health conditions, which may cause underestimation of the true association.^43^ The results are consistent with existing literature that highlights the mental health disparities in care between sexual minority and heterosexual populations.^44,45,46,47^ Specifically, the 2014-2015 Behavioral Risk Factor Surveillance System (308 456 individuals) reported higher odds of depression in gay men compared with heterosexual men (AOR, 2.91; 95% CI, 2.42-3.50) and in lesbian women compared with heterosexual women (AOR, 1.93; 95% CI, 1.60-2.33).^48^ The National Epidemiologic Survey on Alcohol and Related Conditions (34 157 individuals) showed higher prevalence of generalized anxiety (AOR, 2.2; 95% CI, 1.9-2.4) and major depressive disorder (AOR, 1.9; 95% CI, 1.8-2.1) in lesbian, gay, and bisexual individuals compared with heterosexual individuals.^49^ While these studies offer insights into mental health disparities between sexual minority and heterosexual populations, they do not account for disparities by gender identity. Our study addresses these gaps by comparing non-SGM and SGM subgroups in a large national cohort including diverse racial and ethnic groups.

Our analysis revealed that gender diverse people of any sexual orientation assigned female sex at birth show significantly higher odds of PTSD (AOR, 3.67; 95% CI, 2.99-4.50), anxiety (AOR, 2.31; 95% CI, 2.01-2.66), and depression (AOR, 2.59; 95% CI, 2.24-2.98) compared with cisgender heterosexual men. This may be attributed to increased exposure to traumatic experiences such as discrimination, rejection, and the cumulative stress of navigating a society that often fails to recognize or validate gender diverse identities.^50,51^ Our findings highlight the need for targeted interventions to address the unique stressors of gender diverse people of any sexual orientation assigned female sex at birth, underscoring the broad impact of societal stigma, discrimination, and minority stress on mental health, and support systems tailored to gender diverse individuals.^52,53^

It is crucial to clarify that the associations observed between SGM status and adverse mental health outcomes do not imply causality and should not be used to stigmatize these communities. Based on the body of research documenting the adverse impacts of minority stress on SGM individuals, driven by organizational, cultural, and societal stigma and discrimination, it is likely that these factors underlie the marked mental health disparities we observed associated with SGM status, including limited legal protections, exposure to violence, lack of access to gender-affirming treatments, and weaker social support systems.^34,35,54^ These factors not only affect mental health directly by increasing stress and dysregulating neuroendocrine functions but also compromise immune responses, heightening the risk of mental health issues over time.^55^ Our findings provide evidence for the need to tailor mental health interventions for different sexual orientation and gender identity groups.

### Strengths and Limitations

Our study presents several strengths. To the best of our knowledge, it includes the largest sample of SGM participants in a US national cohort, featuring detailed records of sexual orientation and gender identity. This extensive dataset allows us to identify and analyze mental health inequalities across distinct SGM subgroups. To address outcome misclassification, we performed sensitivity analyses with various diagnostic definitions and applied primary and inverse probability weighting regressions to handle confounding. These methods helped evaluate misclassification risk and enhance the validity of our conclusions.

The study also has several limitations. First, while the All of Us Research Program is a diverse cohort, the participants are not nationally representative. The program aims to oversample historically underrepresented populations in biomedical research, including SGM people, enabling more nuanced and granular analysis by SGM subgroup, as demonstrated in this report with the analysis of 6 distinct SGM subgroups. Second, while the study assesses the identity dimension of sexual orientation, it does not assess 2 other important dimensions, specifically attractions and sex and/or gender of sexual partner, which may have different associations with mental health. Third, although we used EHR to assess mental health outcomes, the validity and reliability of this method have not been formally tested. We addressed this by conducting sensitivity analyses using additional definitions of mental health outcomes, and future studies could further investigate the accuracy of these EHR-based classifications. Fourth, the estimates for ASD, eating disorders, and OCD are not robust due to the small sample size in the SGM subgroups, despite the overall large cohort size. Similarly, due to sample size limitations, sexual orientation identity subgroups could not be analyzed separately, though there may be important heterogeneity across minority sexual orientation and identity subgroups. Continued recruitment may provide greater statistical power to explore these mental health disparities comprehensively. Fifth, the present study did not explore mental health disparities through an intersectional lens incorporating SGM status, race and ethnicity, and socioeconomic status, due to limited statistical power. Sixth, some mental health conditions are chronic, and their impact fluctuates due to life events (eg, family death, HIV, or cancer diagnosis), which cross-sectional analysis cannot capture. Future research should explore these aspects by expanding the participant base and gathering longitudinal data.

## Conclusions

In this cross-sectional study of the All of Us Research Program, significant mental health disparities were found between SGM and non-SGM groups, with notable variations observed within SGM subgroups when compared with their cisgender heterosexual counterparts. This study provides evidence of significant mental health disparities within the SGM community, highlighting the importance of targeted interventions to address these inequalities.

## Notes

Supplement 1. **eTable 1.** Systematized Nomenclature of Medicine: Clinical Terms (SNOMED CT) Codes of Mental Health Conditions **eTable 2.** Survey Responses and Data Generalizations for Sexual Orientation, Gender Identity, and Sex Assigned at Birth by Comparison Group in the All of Us Research Program (2017-2022) **eTable 3.** Characteristics of Participants Included and Excluded From the Analysis in the All of Us Research Program (2017-2022) **eTable 4.** Odds Ratios of Mental Health Conditions Between Cisgender Sexual Minority Men and Cisgender Heterosexual Men in the All of Us Research Program (2017-2022) **eTable 5.** Odds Ratios of Mental Health Conditions Between Cisgender Sexual Minority Women and Cisgender Heterosexual Women in the All of Us Research Program (2017-2022) **eTable 6.** Odds Ratios of Mental Health Conditions Between Gender Diverse People Assigned Female at Birth of Any Sexual Orientation and Cisgender Heterosexual Men in the All of Us Research Program (2017-2022) **eTable 7.** Odds Ratios of Mental Health Conditions Between Gender Diverse People Assigned Female at Birth of Any Sexual Orientation and Cisgender Heterosexual Women in the All of Us Research Program (2017-2022) **eTable 8.** Odds Ratios of Mental Health Conditions Between Gender Diverse People Assigned Male at Birth of Any Sexual Orientation and Cisgender Heterosexual Men in the All of Us Research Program (2017-2022) **eTable 9.** Odds Ratios of Mental Health Conditions Between Gender Diverse People Assigned Male at Birth of Any Sexual Orientation and Cisgender Heterosexual Women in the All of Us Research Program (2017-2022) **eTable 10.** Odds Ratios of Mental Health Conditions Between Transgender Men of Any Sexual Orientation and Cisgender Heterosexual Men in the All of Us Research Program (2017-2022) **eTable 11.** Odds Ratios of Mental Health Conditions Between Transgender Women of Any Sexual Orientation and Cisgender Heterosexual Women in the All of Us Research Program (2017-2022) **eTable 12.** Rate Ratios of Mental Health Condition Counts Comparing SGM and Non-SGM Groups in the All of Us Research Program (2017-2022) **eTable 13.** Prescription Names Used with EHR Diagnosed Records to Determine Mental Health Conditions in the All of Us Research Program (2017-2022) **eTable 14.** Sensitivity Analysis of Mental Health Conditions Determined by Both EHR-Diagnosed Codes and Prescription Records by Sexual Orientation and Gender Identity Group in the All of Us Research Program (2017-2022) **eTable 15.** Sensitivity Analysis of Mental Health Conditions Determined by Both EHR-Diagnosed Codes and Prescription Records: Odds Ratios for Mental Health Conditions Comparing SGM and Non-SGM Groups in the All of Us Research Program (2017-2022) **eTable 16.** Sensitivity Analysis of Mental Health Conditions Determined by Both EHR-Diagnosed Codes and Prescription Records: Rate Ratios for Mental Health Condition Counts Comparing SGM and Non-SGM Groups in the All of Us Research Program (2017-2022) **eTable 17.** EHR Visit Types and Data Generalizations for Classifying Inpatient and Outpatient Visits in the All of Us Research Program (2017-2022) **eTable 18.** Sensitivity Analysis of Mental Health Conditions Determined by Both EHR Diagnosed Codes and Inpatient and Outpatient Records by Sexual Orientation and Gender Identity Group in the All of Us Research Program (2017-2022) **eTable 19.** Sensitivity Analysis of Mental Health Conditions Determined by Both EHR Diagnosed Codes and Inpatient Outpatient Records: Odds Ratios for Mental Health Conditions Comparing SGM and Non-SGM Groups in the All of Us Research Program (2017-2022) **eTable 20.** Sensitivity Analysis of Mental Health Conditions Determined by Both EHR Diagnosed Codes and Inpatient and Outpatient Records: Rate Ratios for Mental Health Condition Counts Comparing SGM and Non-SGM Groups in the All of Us Research Program (2017-2022) **eTable 21.** Characteristics of Intersex Participants With and Without Electronic Health Records the All of Us Research Program (2017-2022) **eTable 22.** Electronic Health Record Diagnosed Mental Health Intersex Participants With Any Sexual Orientation in All of Us Research Program (2017-2022) **eFigure 1.** Percentage of Missing Electronic Health Record Data by Sexual Orientation and Gender Identity Groups in the All of Us Research Program (2017-2022) **eFigure 2.** Age Distribution Across Subgroups and Post Hoc Analysis in the All of Us Research Program (2017-2022) **eFigure 3.** Income Distribution Across Subgroups and Post Hoc Analysis in the All of Us Research Program (2017-2022) **eFigure 4.** Association Between Variables Used in the All of Us Research Program Analysis (2017-2022) **eFigure 5.** Absolute Standardized Mean Differences Between Subgroups: Part 1 **eFigure 6.** Absolute Standardized Mean Differences Between Subgroups: Part 2 **eFigure 7.** Propensity Score Distribution Between Sexual and Gender Minority Groups (SGM) Compared With Their Non-SGM Counterparts **eFigure 8.** Rate Ratios of Mental Health Condition Counts Comparing SGM and Non-SGM Groups in the All of Us Research Program (2017-2022) **eFigure 9.** Sensitivity Analysis of Mental Health Conditions Determined by Both EHR Diagnosed Codes and Prescription Records: Rate Ratios for Mental Health Condition Counts Comparing SGM and Non-SGM Groups in the All of Us Research Program (2017-2022) **eFigure 10.** Sensitivity Analysis of Mental Health Conditions Determined by Both EHR Diagnosed Codes and Inpatient and Outpatient Records: Rate Ratios for Mental Health Condition Counts Comparing SGM and Non-SGM Groups in the All of Us Research Program (2017-2022) **eFigure 11.** Sensitivity Analysis on Adjusted Odds Ratios of Mental Health Conditions Among Sexual and Gender Minority Participants in the All of Us Research Program (2017-2022): Part 1 **eFigure 12.** Sensitivity Analysis on Adjusted Odds Ratios of Mental Health Conditions Among Sexual and Gender Minority Participants in the All of Us Research Program (2017-2022): Part 2
eReferences.

Supplement 2.
Data Sharing Statement
