# Supplementary material for: Mental Health Disparities by Sexual Orientation and Gender Identity in the All of Us Research Program
Source: JAMA Netw Open. 2025 Jan 29;8(1):e2456264. doi: 10.1001/jamanetworkopen.2024.56264 (PMC11780479; doi:10.1001/jamanetworkopen.2024.56264)
Supplement: Supplement 1. — eTable 1. Systematized Nomenclature of Medicine: Clinical Terms (SNOMED CT) Codes of Mental Health Conditions eTable 2. Survey Responses and Data Generalizations for Sexual Orientation, Gender Identity, and Sex Assigned at Birth by Comparison Group in the All of Us Research Program (2017-2022) eTable 3. Characteristics of Participants Included and Excluded From the Analysis in the All of Us Research Program (2017-2022) eTable 4. Odds Ratios of Mental Health Conditions Between Cisgender Sexual Minority Men and Cisgender Heterosexual Men in the All of Us Research Program (2017-2022) eTable 5. Odds Ratios of Mental Health Conditions Between Cisgender Sexual Minority Women and Cisgender Heterosexual Women in the All of Us Research Program (2017-2022) eTable 6. Odds Ratios of Mental Health Conditions Between Gender Diverse People Assigned Female at Birth of Any Sexual Orientation and Cisgender Heterosexual Men in the All of Us Research Program (2017-2022) eTable 7. Odds Ratios of Mental Health Conditions Between Gender Diverse People Assigned Female at Birth of Any Sexual Orientation and Cisgender Heterosexual Women in the All of Us Research Program (2017-2022) eTable 8. Odds Ratios of Mental Health Conditions Between Gender Diverse People Assigned Male at Birth of Any Sexual Orientation and Cisgender Heterosexual Men in the All of Us Research Program (2017-2022) eTable 9. Odds Ratios of Mental Health Conditions Between Gender Diverse People Assigned Male at Birth of Any Sexual Orientation and Cisgender Heterosexual Women in the All of Us Research Program (2017-2022) eTable 10. Odds Ratios of Mental Health Conditions Between Transgender Men of Any Sexual Orientation and Cisgender Heterosexual Men in the All of Us Research Program (2017-2022) eTable 11. Odds Ratios of Mental Health Conditions Between Transgender Women of Any Sexual Orientation and Cisgender Heterosexual Women in the All of Us Research Program (2017-2022) eTable 12. Rate Ratios of Mental Health Condition C [file jamanetwopen-e2456264-s001.pdf]

Supplementary Online Content

Lu JA, Soltani S, Austin SB, Rehkoph DH, Lunn MR, Langston ME. Mental health disparities by sexual orientation and gender identity in All of Us Research Program. *JAMA Netw Open*. 2025;8(1):e2456264. doi:10.1001/jamanetworkopen.2024.56264

eTable 1. Systematized Nomenclature of Medicine: Clinical Terms (SNOMED CT) Codes of Mental Health Conditions

eTable 2. Survey Responses and Data Generalizations for Sexual Orientation, Gender Identity, and Sex Assigned at Birth by Comparison Group in the All of Us Research Program (2017-2022)

eTable 3. Characteristics of Participants Included and Excluded From the Analysis in the All of Us Research Program (2017-2022)

eTable 4. Odds Ratios of Mental Health Conditions Between Cisgender Sexual Minority Men and Cisgender Heterosexual Men in the All of Us Research Program (2017-2022)

eTable 5. Odds Ratios of Mental Health Conditions Between Cisgender Sexual Minority Women and Cisgender Heterosexual Women in the All of Us Research Program (2017-2022)

eTable 6. Odds Ratios of Mental Health Conditions Between Gender Diverse People Assigned Female at Birth of Any Sexual Orientation and Cisgender Heterosexual Men in the All of Us Research Program (2017-2022)

eTable 7. Odds Ratios of Mental Health Conditions Between Gender Diverse People Assigned Female at Birth of Any Sexual Orientation and Cisgender Heterosexual Women in the All of Us Research Program (2017-2022)

eTable 8. Odds Ratios of Mental Health Conditions Between Gender Diverse People Assigned Male at Birth of Any Sexual Orientation and Cisgender Heterosexual Men in the All of Us Research Program (2017-2022)

eTable 9. Odds Ratios of Mental Health Conditions Between Gender Diverse People Assigned Male at Birth of Any Sexual Orientation and Cisgender Heterosexual Women in the All of Us Research Program (2017-2022)

eTable 10. Odds Ratios of Mental Health Conditions Between Transgender Men of Any Sexual Orientation and Cisgender Heterosexual Men in the All of Us Research Program (2017-2022)

eTable 11. Odds Ratios of Mental Health Conditions Between Transgender Women of Any Sexual Orientation and Cisgender Heterosexual Women in the All of Us Research Program (2017-2022)

eTable 12. Rate Ratios of Mental Health Condition Counts Comparing SGM and Non-SGM Groups in the All of Us Research Program (2017-2022)

eTable 13. Prescription Names Used with EHR Diagnosed Records to Determine Mental Health Conditions in the All of Us Research Program (2017-2022)

eTable 14. Sensitivity Analysis of Mental Health Conditions Determined by Both EHR-Diagnosed Codes and Prescription Records by Sexual Orientation and Gender Identity Group in the All of Us Research Program (2017-2022)

eTable 15. Sensitivity Analysis of Mental Health Conditions Determined by Both EHR-Diagnosed Codes and Prescription Records: Odds Ratios for Mental Health Conditions Comparing SGM and Non-SGM Groups in the All of Us Research Program (2017-2022)

eTable 16. Sensitivity Analysis of Mental Health Conditions Determined by Both EHR-Diagnosed Codes and Prescription Records: Rate Ratios for Mental Health Condition Counts Comparing SGM and Non-SGM Groups in the All of Us Research Program (2017-2022)

© 2025 Lu JA et al. *JAMA Network Open*.

eTable 17. EHR Visit Types and Data Generalizations for Classifying Inpatient and Outpatient Visits in the All of Us Research Program (2017-2022)

eTable 18. Sensitivity Analysis of Mental Health Conditions Determined by Both EHR Diagnosed Codes and Inpatient and Outpatient Records by Sexual Orientation and Gender Identity Group in the All of Us Research Program (2017-2022)

eTable 19. Sensitivity Analysis of Mental Health Conditions Determined by Both EHR Diagnosed Codes and Inpatient Outpatient Records: Odds Ratios for Mental Health Conditions Comparing SGM and Non-SGM Groups in the All of Us Research Program (2017-2022)

eTable 20. Sensitivity Analysis of Mental Health Conditions Determined by Both EHR Diagnosed Codes and Inpatient and Outpatient Records: Rate Ratios for Mental Health Condition Counts Comparing SGM and Non-SGM Groups in the All of Us Research Program (2017-2022)

eTable 21. Characteristics of Intersex Participants With and Without Electronic Health Records the All of Us Research Program (2017-2022)

eTable 22. Electronic Health Record Diagnosed Mental Health Intersex Participants With Any Sexual Orientation in All of Us Research Program (2017-2022)

eFigure 1. Percentage of Missing Electronic Health Record Data by Sexual Orientation and Gender Identity Groups in the All of Us Research Program (2017-2022)

eFigure 2. Age Distribution Across Subgroups and Post Hoc Analysis in the All of Us Research Program (2017-2022)

eFigure 3. Income Distribution Across Subgroups and Post Hoc Analysis in the All of Us Research Program (2017-2022)

eFigure 4. Association Between Variables Used in the All of Us Research Program Analysis (2017-2022)

eFigure 5. Absolute Standardized Mean Differences Between Subgroups: Part 1

eFigure 6. Absolute Standardized Mean Differences Between Subgroups: Part 2

eFigure 7. Propensity Score Distribution Between Sexual and Gender Minority Groups (SGM) Compared With Their Non-SGM Counterparts

eFigure 8. Rate Ratios of Mental Health Condition Counts Comparing SGM and Non-SGM Groups in the All of Us Research Program (2017-2022)

eFigure 9. Sensitivity Analysis of Mental Health Conditions Determined by Both EHR Diagnosed Codes and Prescription Records: Rate Ratios for Mental Health Condition Counts Comparing SGM and Non-SGM Groups in the All of Us Research Program (2017-2022)

eFigure 10. Sensitivity Analysis of Mental Health Conditions Determined by Both EHR Diagnosed Codes and Inpatient and Outpatient Records: Rate Ratios for Mental Health Condition Counts Comparing SGM and Non-SGM Groups in the All of Us Research Program (2017-2022)

eFigure 11. Sensitivity Analysis on Adjusted Odds Ratios of Mental Health Conditions Among Sexual and Gender Minority Participants in the All of Us Research Program (2017-2022): Part 1

eFigure 12. Sensitivity Analysis on Adjusted Odds Ratios of Mental Health Conditions Among Sexual and Gender Minority Participants in the All of Us Research Program (2017-2022): Part 2

eReferences.

This supplementary material has been provided by the authors to give readers additional information about their work.

© 2025 Lu JA et al. *JAMA Network Open*.

eTable 1. Systematized Nomenclature of Medicine: Clinical Terms (SNOMED CT) Codes of Mental Health Conditions

Mental Health Condition	SNOMED CT Name	SNOMED CT Code
Anxiety	Anxiety	48694002
ADHD	Attention deficit hyperactivity disorder	406506008
ASD	Autism spectrum disorder	35919005
Bipolar disorder	Bipolar disorder	13746004
Eating disorder	Eating disorder	72366004
Depression	Depressive disorder	35489007
OCD	Obsessive-compulsive disorder	191736004
Personality disorder	Personality disorder	33449004
PTSD	Posttraumatic stress disorder	47505003
Schizophrenia	Schizophrenia	58214004

Abbreviations: ADHD, Attention-deficit/hyperactivity disorder; ASD, Autism spectrum disorder; OCD, Obsessive-compulsive disorder; PTSD, Post-traumatic stress disorder.

© 2025 Lu JA et al. *JAMA Network Open*.

eTable 2. Survey Responses and Data Generalizations for Sexual Orientation, Gender Identity, and Sex Assigned at Birth by Comparison Group in the All of Us Research Program (2017-2022)

**Comparison Group**	**Gender Identity Survey Response [a]**	**Sex Assigned at Birth Survey Response [b]**	**Sexual Orientation Survey Response [c]**
Cisgender heterosexual women	"Woman" only	Female	• "Straight; that is not gay or lesbian, etc." only
Cisgender heterosexual men	"Man" only	Male	• "Straight; that is not gay or lesbian, etc." only
Cisgender sexual minority women	"Woman" only	Female	Anyone who answered the sexual orientation item (i.e., not skipped, not missing) AND answered anything other than the following answer choices alone or in any combination with each other:<br>• "Straight; that is not gay or lesbian, etc."<br>• "Have not figured out or are in the process of figuring out your sexuality"<br>• "Do not think of yourself as having sexuality"<br>• "Do not use labels to identify yourself"<br>• "Don't know the answer"<br>• "Prefer not to answer"
Cisgender sexual minority men	"Man" only	Male	Anyone who answered the sexual orientation item (i.e., not skipped, not missing) AND answered anything other than the following answer choices alone or in any combination with each other:<br>• "Straight; that is not gay or lesbian, etc."<br>• "Have not figured out or are in the process of figuring out your sexuality"<br>• "Do not think of yourself as having sexuality"<br>• "Do not use labels to identify yourself"<br>• "Don't know the answer"<br>• "Prefer not to answer"
Gender-diverse people assigned male sex at birth (of any sexual orientation)	Anyone who answered the gender identity item (i.e., not skipped, not missing) AND did not select "prefer not to answer" only AND does not fit into any of the other groups	Male	Any
Gender-diverse people assigned female sex at birth (of any sexual orientation)	Anyone who answered the gender identity item (i.e., not skipped, not missing) AND did not select "prefer not to answer" only AND	Female	Any

© 2025 Lu JA et al. *JAMA Network Open*.

Comparison Group	Gender Identity Survey Response [a]	Sex Assigned at Birth Survey Response [b]	Sexual Orientation Survey Response [c]
	does not fit into any of the other groups		
Transgender men (of any sexual orientation	• "Man" only<br>• "Transgender" only<br>• "Trans man/Transgender Man/FTM" only<br>Any combination of the 3 selections above	Female	Any
Transgender women (of any sexual orientation	• "Woman" only<br>• "Transgender" only<br>• "Trans woman/Transgender Woman/MTF" only<br>Any combination of the 3 selections above	Male	Any

a. Gender identity was measured by the item, "What terms best express how you describe your gender identity?" Participants could select any (or multiple) responses including man, woman, non-binary, transgender, none of these describe me and I'd like to consider additional options, and/or prefer not to answer. Participants who endorsed non-binary, transgender, or none of these describe me and I'd like to consider additional options were shown the following response options: trans man/transgender man/FTM, trans woman/transgender women/MTF, genderqueer, genderfluid, gender variant, Two-spirit, questioning or unsure of gender identity, and/or none of these describe me, and I want to specify.
b. Sex assigned at birth was measured by the item, "What was your biological sex assigned at birth?" Participants were excluded if they answered "intersex," "prefer not to answer," or skipped the question.
c. Sexual orientation was measured by the item, "Which of the following best represents how you think of yourself?" Participants could select any (or multiple) responses including gay, lesbian, straight; that is, not gay or lesbian, etc., bisexual, and/or none of these describe me and I'd like to consider additional options. Participants who endorsed none of these describe me and I'd like to consider additional options were shown the following responses: queer; polysexual, omnisexual, sapiosexual or pansexual; asexual; Two-spirit; have not figure out or in the process of figuring out your sexuality; mostly straight, but sometimes attracted to people of your own sex; do not think of yourself as having sexuality; do not use labels to identity yourself; don't know the answer; no I mean something else, please specify; and/or prefer not to answer.

© 2025 Lu JA et al. *JAMA Network Open*.

eFigure 1. Percentage of Missing Electronic Health Record Data by Sexual Orientation and Gender Identity Groups in the All of Us Research Program (2017-2022)

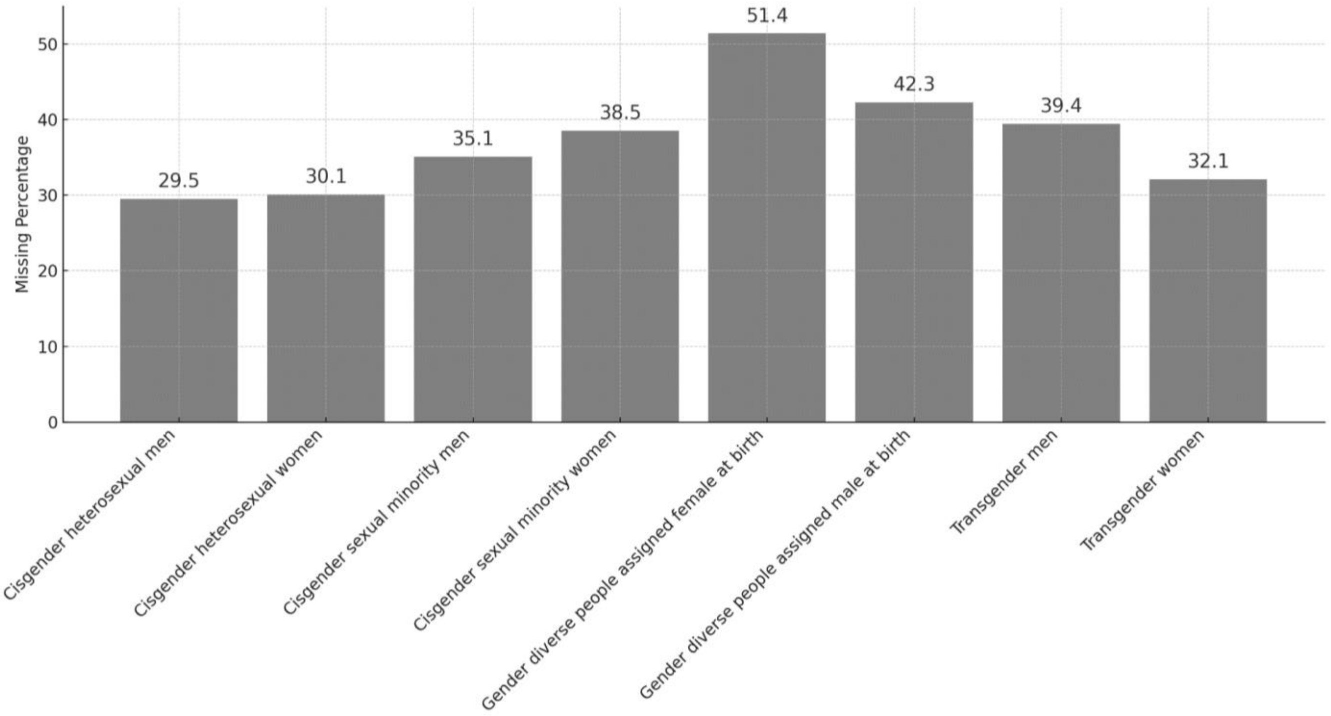

© 2025 Lu JA et al. *JAMA Network Open*.

eTable 3. Characteristics of Participants Included and Excluded From the Analysis in the All of Us Research Program (2017-2022)

	No. (%)			Standardized Mean Difference [b]
Categorical variables	Overall cohort n = 413 457	Participants included n = 269 947	Participants excluded n = 143 510	
**Race and ethnicity** [a]				0.037
African American or Black	82 196 (19.9)	56 126 (20.8)	26 070 (18.2)	
Asian	17 080 (4.1)	9587 (3.6)	7493 (5.2)	
Hispanic or Latinx	74 114 (17.9)	50 836 (18.8)	23 278 (16.2)	
Middle Eastern or North African	4333 (1.0)	2751 (1.0)	1582 (1.1)	
Native Hawaiian or other Pacific Islander	1112 (0.3)	677 (0.3)	435 (0.3)	
White	236 162 (57.1)	154 260 (57.1)	81 902 (57.1)	
**Annual Household Income** [a]				0.027
Less than $25,000	102 719 (24.8)	69 637 (25.8)	33 082 (23.1)	
$25,000-$49,999	61 819 (15.0)	40 736 (15.1)	21 083 (14.7)	
$50,000-$99,999	77 496 (18.7)	50 141 (18.6)	27 355 (19.1)	
$100,000-$149,999	41 789 (10.1)	26 664 (9.9)	15 125 (10.5)	
≥$150,000	46 526 (11.3)	29 491 (10.9)	17 035 (11.9)	
Prefer to not answer or skipped	83 011 (20.1)	53 278 (19.7)	29 733 (20.7)	
**Education levels**				0.085
High school graduate or less	113 446 (27.4)	78 328 (29.0)	35 118 (24.5)	
Some college	104 136 (25.2)	69 946 (25.9)	34 190 (23.8)	
4-year College graduate	93 238 (22.6)	60 680 (22.5)	32 558 (22.7)	
Advanced degree	89 107 (21.6)	56 174 (20.8)	32 933 (23.0)	
Prefer not to answer or skipped	13 433 (3.2)	4819 (1.8)	8614 (6.0)	
**Employed for wages**				0.134
No	225 403 (54.5)	154 018 (57.1)	71 385 (49.8)	
Yes	17 1730 (41.5)	108 998 (40.4)	62 732 (43.7)	
Prefer not to answer or skipped	16 227 (3.9)	6931 (2.6)	9296 (6.5)	
**Owned a home**				0.083
No	202 322 (48.9)	134 411 (49.8)	67 911 (47.4)	
Yes	189 576 (45.9)	125 229 (46.4)	64 347 (44.9)	
Prefer not to answer or skipped	21 462 (5.2)	10 307 (3.8)	11 155 (7.8)	
**Insured**				0.139
No	26 779 (6.5)	15 433 (5.7)	11 346 (8.0)	
Yes	37 1633 (90.3)	248 614 (92.1)	123 019 (86.9)	
Prefer not to answer or skipped	13 158 (3.2)	5900 (2.2)	7272 (5.1)	
**Enrollment year**				0.092
2017	10 504 (2.5)	8066 (3.0)	2438 (1.7)	
2018	999 27 (24.2)	67 318 (24.9)	32 609 (22.7)	
2019	16 4249 (39.7)	112 662 (41.7)	51 587 (35.9)	
2020	49 965 (12.1)	29 551 (10.9)	20 414 (14.2)	
2021	48 795 (11.8)	30 101 (11.2)	18 694 (13.0)	

© 2025 Lu JA et al. *JAMA Network Open*.

	No. (%)			Standardized Mean Difference [b]
Categorical variables	Overall cohort<br>n = 413 457	Participants included<br>n = 269 947	Participants excluded<br>n = 143 510	
2022	400 16 (9.7)	22 249 (8.2)	17 767 (12.4)	
Continuous variables				
Age, mean (SD)	55.8 (17.1)	56.9 (17.0)	53.9 (17.1)	0.176
Age, median (IQR)	57.0 (41-69)	59.0 (43-70)	55.0 (39-68)	

Abbreviations: SD: standard deviation; IQR: Interquartile range

a. Categories are not mutually exclusive; they do not sum to the column total because participants may self-identify in multiple groups.
b. A standardized mean difference (SMD) values below 0.2 are considered to represent small differences between the two groups.[1]

© 2025 Lu JA et al. *JAMA Network Open*.

eFigure 2. Age Distribution Across Subgroups and Post Hoc Analysis in the All of Us Research Program (2017-2022)

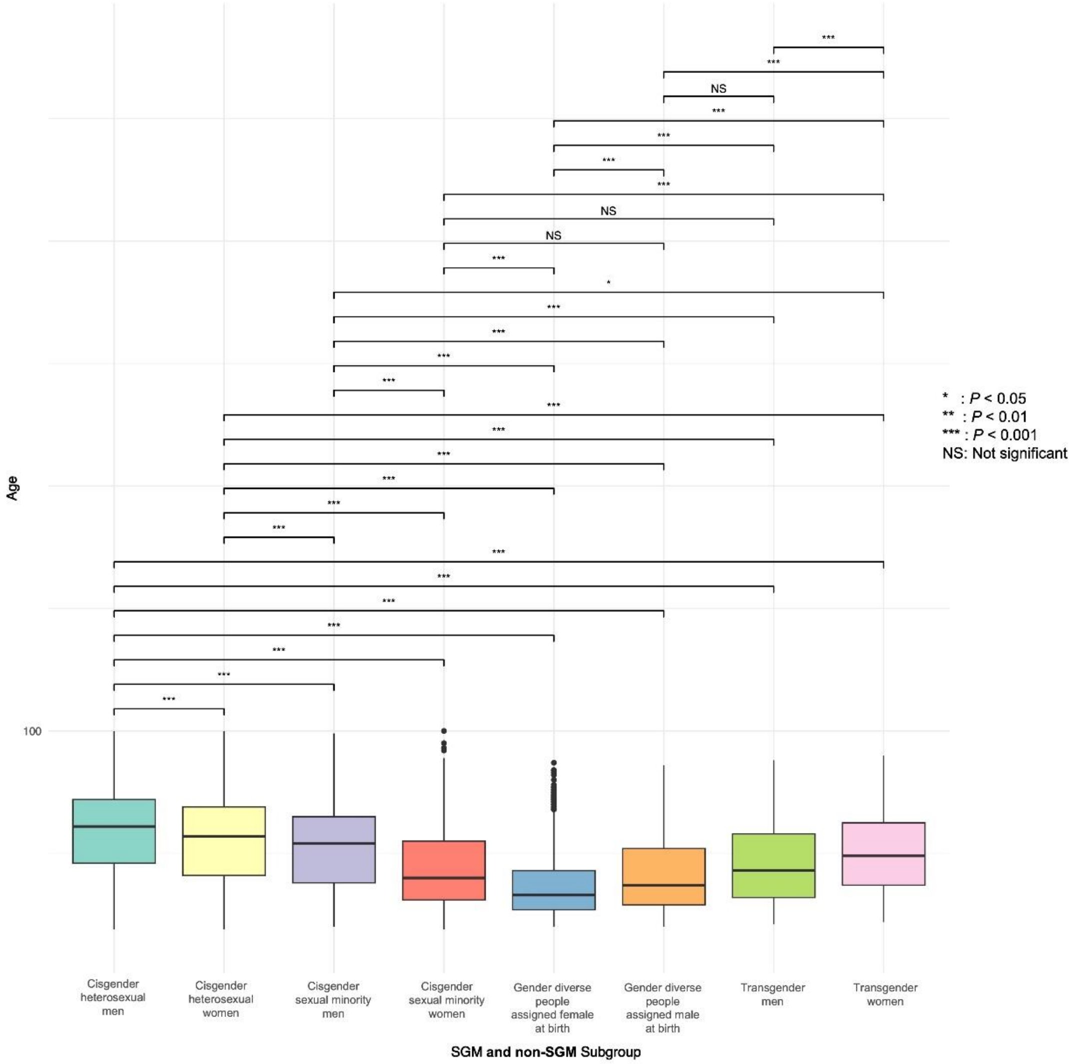

Note: The Kruskal-Wallis test was used to compare medians across multiple groups, showing statistical significance with H=10472, df=7, $P < 0.001$. Dunn's test was then performed as a post-hoc analysis for pairwise comparisons, with Bonferroni correction applied to adjust for multiple comparisons.

© 2025 Lu JA et al. *JAMA Network Open*.

eFigure 3. Income Distribution Across Subgroups and Post Hoc Analysis in the All of Us Research Program (2017-2022)

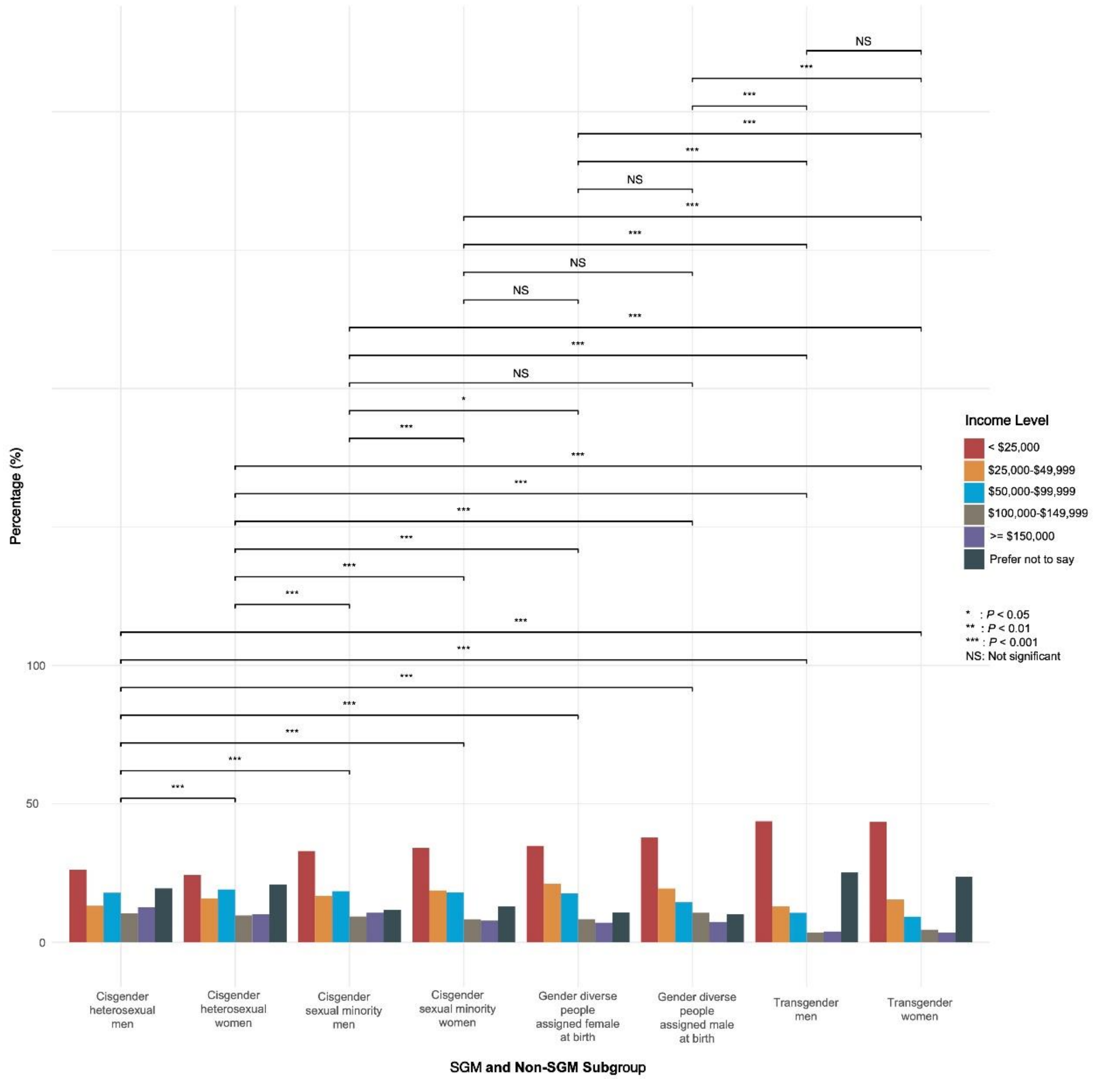

Note: Chi-Square Test for Homogeneity was conducted to compare proportions across multiple groups, which showed statistical significance with χ^2=2712.8, df = 35, P < 0.001. Pairwise comparisons of proportions were performed, again using Bonferroni correction for multiple comparisons.

© 2025 Lu JA et al. *JAMA Network Open*.

eFigure 4. Association Between Variables Used in the All of Us Research Program Analysis (2017-2022)

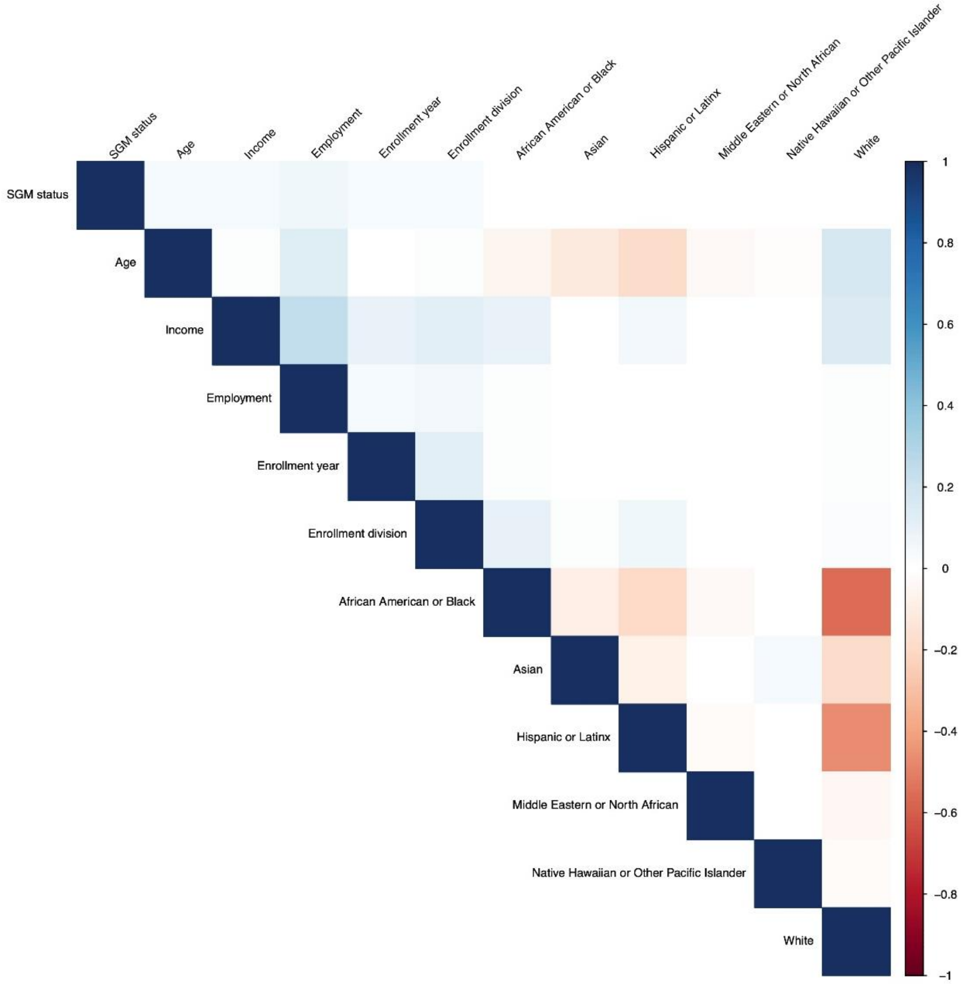

Note: Three different association measures were used based on variable types: Pearson correlation was used for relationships between numeric variable (age) and all race variables (treated as binary), as well as between different race variables themselves, with values ranging from -1 to +1; Cramér's V was calculated for associations between categorical variables with three or more categories (SGM status, income, employment status, enrollment year, enrollment division), with values ranging from 0 to 1; eta-squared was calculated to measure the proportion of variance explained when examining relationships between numeric/binary variables and categorical variables (e.g., age with SGM status, or binary race and ethnicity variables with employment status), with values ranging from 0 to 1.

© 2025 Lu JA et al. *JAMA Network Open*.

eFigure 5. Absolute Standardized Mean Differences Between Subgroups: Part 1

A) Cisgender Sexual Minority Men Compared to Cisgender Heterosexual Men, B) Cisgender Sexual Minority Women Compared to Cisgender Heterosexual Women, C) Gender Diverse People Assigned Female at Birth (of Any Sexual Orientation) Compared to Cisgender Heterosexual Men, and D) Gender Diverse People Assigned Female at Birth (of Any Sexual Orientation) Compared to Cisgender Heterosexual Women

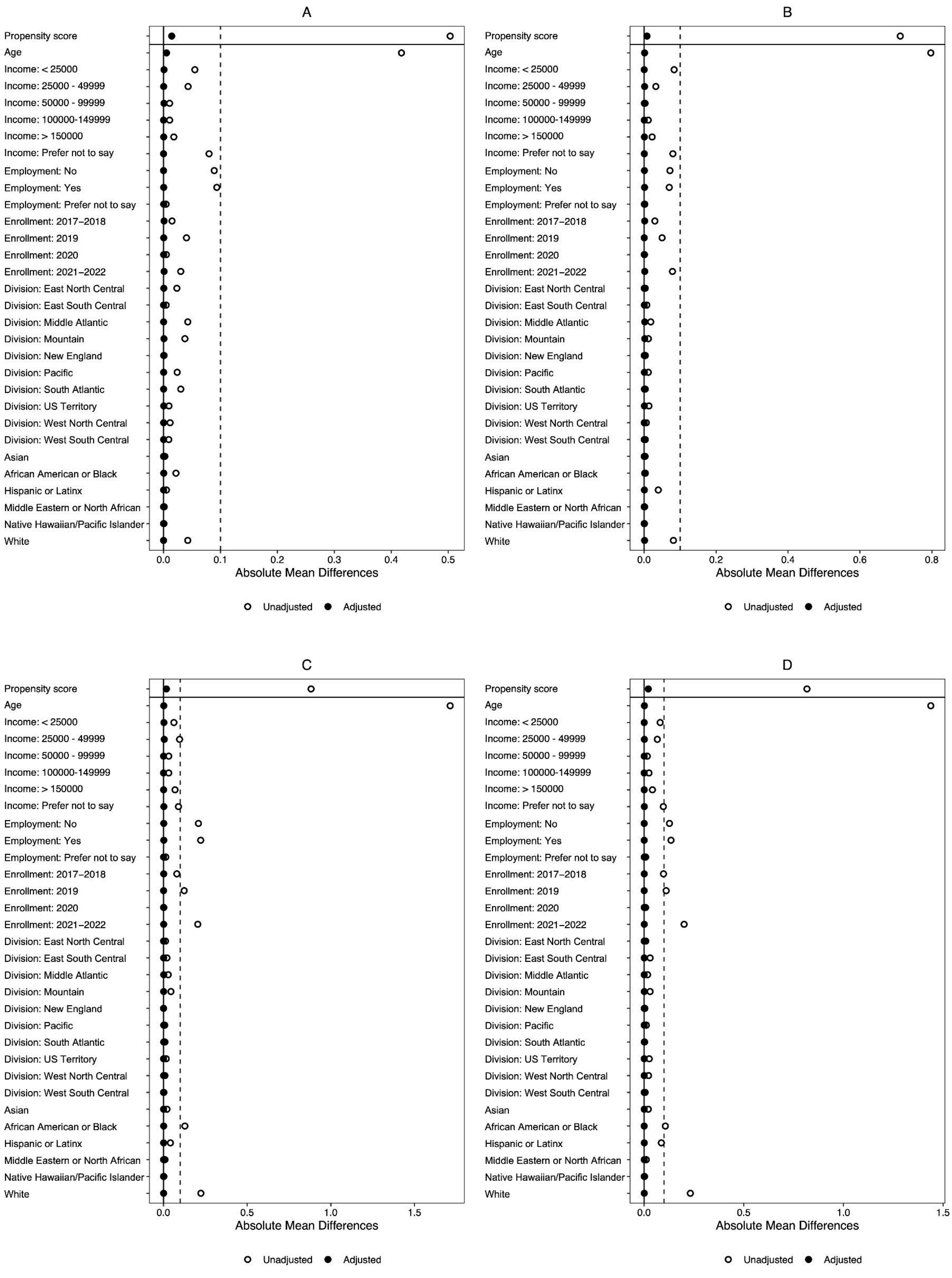

Note: The dashed line marks an absolute mean difference (AMD) of 0.1 (or 10%), a common threshold for determining covariate balance after weighting. If the absolute AMD for a covariate is less than 0.1, the covariate is considered well-balanced.

© 2025 Lu JA et al. *JAMA Network Open*.

eFigure 6. Absolute Standardized Mean Differences Between Subgroups: Part 2

E) Gender Diverse People Assigned Male at Birth (of Any Sexual Orientation) Compared to Cisgender Heterosexual Men, F) Gender Diverse People Assigned Male at Birth (of Any Sexual Orientation) Compared to Cisgender Heterosexual Women, G) Transgender Men (of Any Sexual Orientation) Compared to Cisgender Heterosexual Men, and H) Transgender Women (of Any Sexual Orientation) Compared to Cisgender Heterosexual Women

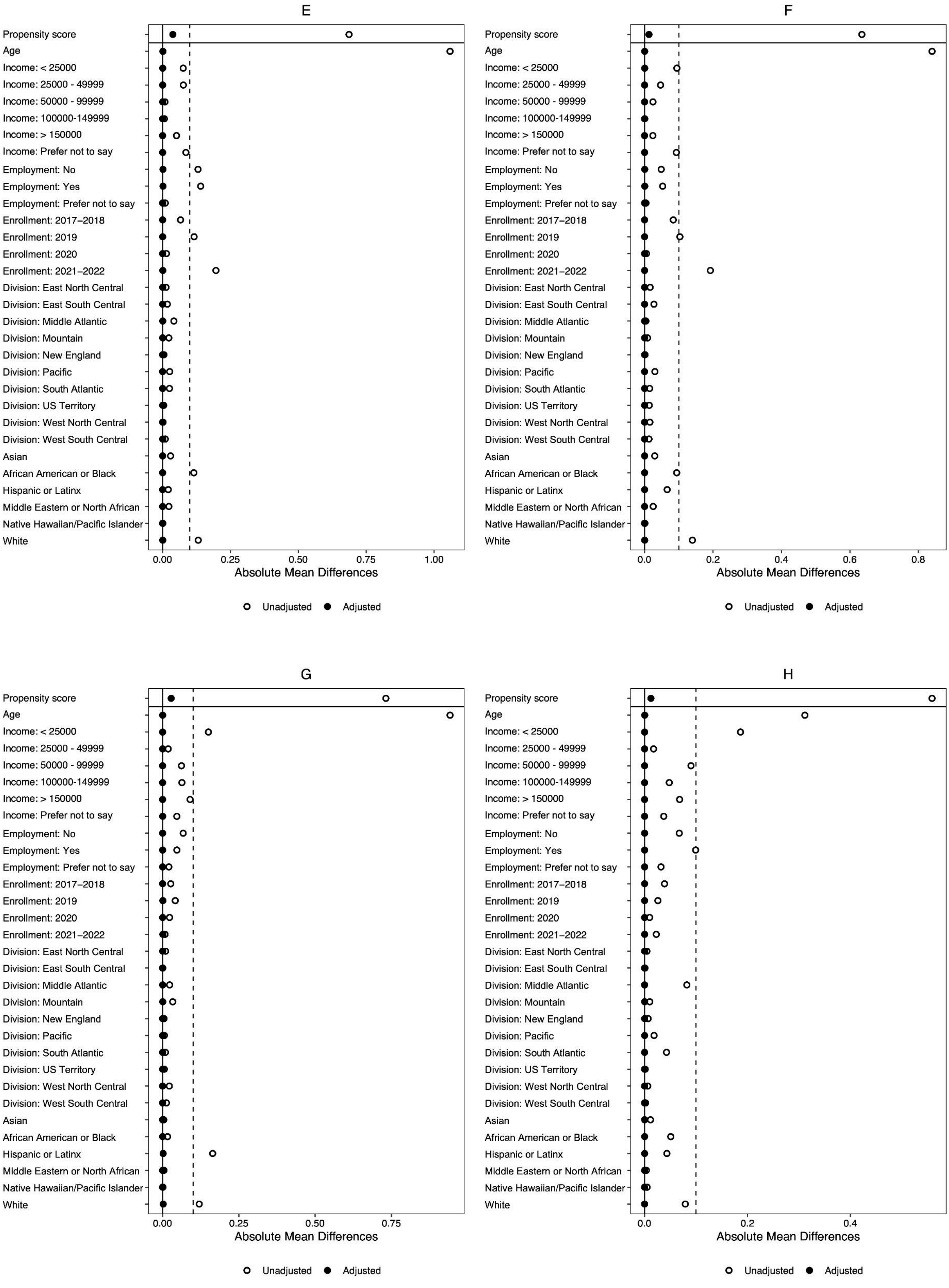

Note: The dashed line marks an absolute mean difference (AMD) of 0.1 (or 10%), a common threshold for determining covariate balance after weighting. If the absolute AMD for a covariate is less than 0.1, the covariate is considered well-balanced

© 2025 Lu JA et al. *JAMA Network Open*.

eFigure 7. Propensity Score Distribution Between Sexual and Gender Minority Groups (SGM) Compared With Their Non-SGM Counterparts

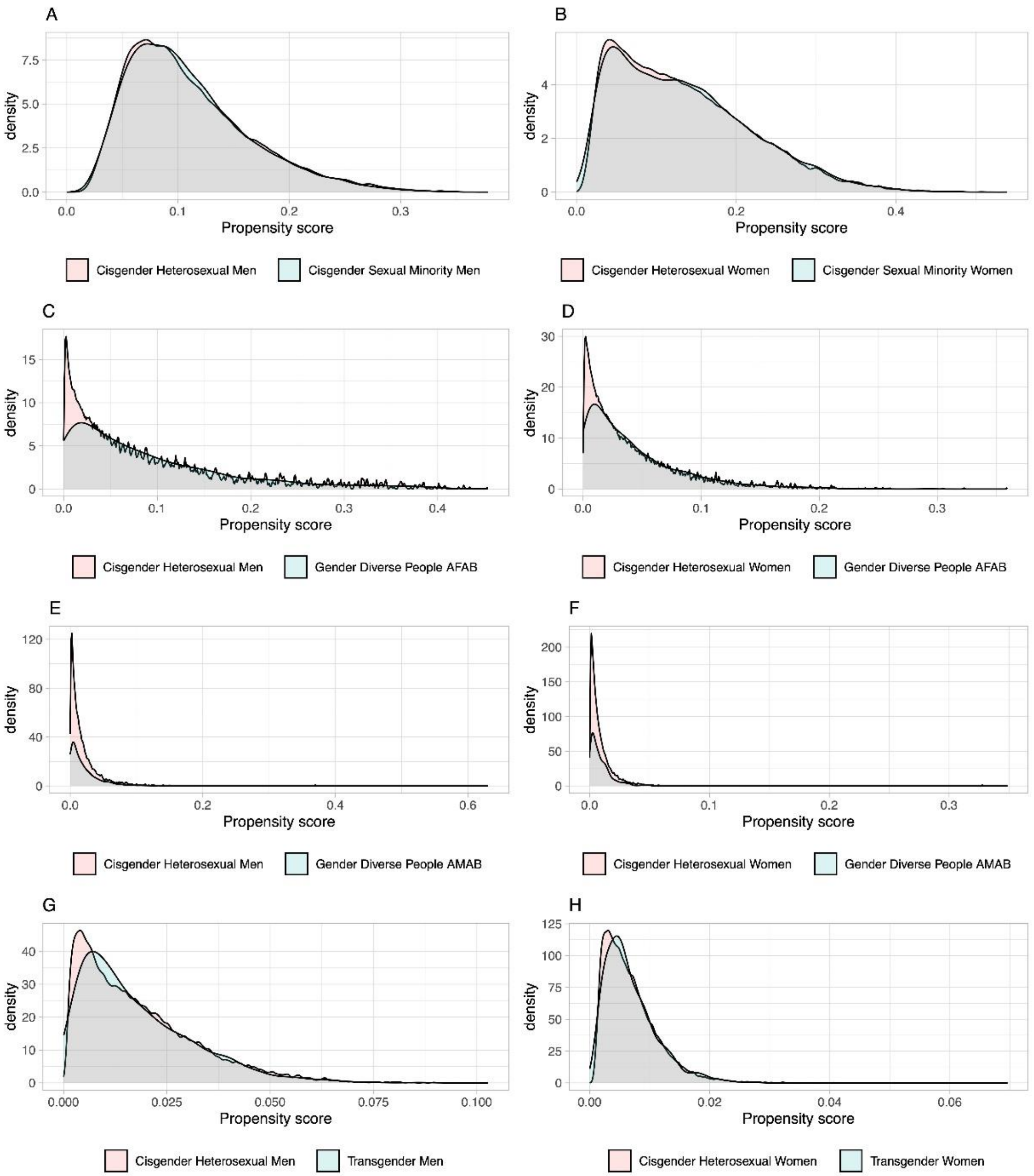

© 2025 Lu JA et al. *JAMA Network Open*.

eTable 4. Odds Ratios of Mental Health Conditions Between Cisgender Sexual Minority Men and Cisgender Heterosexual Men in the All of Us Research Program (2017-2022)

**Mental Health Conditions**	**Unadjusted OR (95% CI)**	**Regression adjusted [a] OR (95% CI)**	**Regression adjusted [b] OR (95% CI)**	**PS weighted [a] OR (95% CI)**
ADHD	1.91 (1.68, 2.17)	1.43 (1.26, 1.62)	1.50 (1.32, 1.70)	1.43 (1.25, 1.62)
Anxiety	1.70 (1.61, 1.80)	1.63 (1.54, 1.72)	1.69 (1.60, 1.79)	1.64 (1.55, 1.73)
ASD	2.33 (1.62, 3.35)	1.47 (1.02, 2.11)	1.57 (1.09, 2.27)	1.47 (1.02, 2.13)
Bipolar disorder	2.11 (1.93, 2.32)	1.87 (1.70, 2.56)	1.93 (1.76, 2.12)	1.88 (1.71, 2.07)
Depression	1.90 (1.80, 2.00)	1.86 (1.77, 1.96)	1.93 (1.83, 2.03)	1.87 (1.77, 1.97)
Eating disorder	1.68 (1.17, 2.41)	1.79 (1.24, 2.59)	1.84 (1.28, 2.65)	1.81 (1.25, 2.60)
OCD	1.92 (1.42, 2.59)	1.56 (1.15, 2.12)	1.70 (1.25, 2.31)	1.55 (1.14, 2.11)
Personality disorder	1.76 (1.50, 2.08)	1.69 (1.43, 2.00)	1.75 (1.49, 2.07)	1.71 (1.45, 2.02)
PTSD	1.36 (1.22, 1.52)	1.37 (1.23, 1.53)	1.38 (1.24, 1.54)	1.39 (1.25, 1.55)
Schizophrenia	1.28 (1.08, 1.52)	1.19 (1.00, 1.41)	1.15 (0.97, 1.36)	1.20 (1.01, 1.42)

Abbreviations: OR: Odds Ratio; CI: Confidence Interval; PS: Propensity Score; ADHD, Attention-deficit/hyperactivity disorder; ASD, Autism spectrum disorder; OCD, Obsessive-compulsive disorder; PTSD, Post-traumatic stress disorder.

a. Models adjusted for current age (continuous), annual income, employment, enrollment year, US census division, and race/ethnicity

b. Models adjusted for current age (continuous), annual income, employment, enrollment year, and US census division

© 2025 Lu JA et al. *JAMA Network Open*.

eTable 5. Odds Ratios of Mental Health Conditions Between Cisgender Sexual Minority Women and Cisgender Heterosexual Women in the All of Us Research Program (2017-2022)

**Mental Health Conditions**	**Unadjusted OR (95% CI)**	**Regression adjusted [a] OR (95% CI)**	**Regression adjusted [b] OR (95% CI)**	**PS weighted [a] OR (95% CI)**
ADHD	2.81 (2.56, 3.08)	1.62 (1.47, 1.79)	1.86 (1.69, 2.05)	1.66 (1.50, 1.83)
Anxiety	1.42 (1.37, 1.48)	1.26 (1.21, 1.31)	1.34 (1.29, 1.40)	1.31 (1.26, 1.36)
ASD	8.95 (6.29, 12.73)	3.72 (2.51, 5.50)	4.43 (3.02, 6.51)	3.40 (2.31, 5.01)
Bipolar disorder	2.86 (2.68, 3.07)	2.09 (1.95, 2.25)	2.28 (2.12, 2.44)	2.25 (2.10, 2.42)
Depression	1.41 (1.35, 1.47)	1.39 (1.33, 1.45)	1.45 (1.39, 1.51)	1.45 (1.39, 1.51)
Eating disorder	1.82 (1.55, 2.14)	1.35 (1.14, 1.59)	1.46 (1.23, 1.72)	1.39 (1.17, 1.64)
OCD	2.20 (1.78, 2.71)	1.52 (1.23, 1.89)	1.73 (1.39, 2.14)	1.67 (1.34, 2.07)
Personality disorder	2.71 (2.42, 3.04)	1.96 (1.73, 2.21)	2.13 (1.89, 2.39)	2.05 (1.82, 2.32)
PTSD	2.71 (2.52, 2.91)	1.85 (1.72, 2.00)	1.95 (1.81, 2.10)	1.99 (1.85, 2.15)
Schizophrenia	2.09 (1.76, 2.50)	1.84 (1.53, 2.21)	1.83 (1.52, 2.20)	1.96 (1.63, 2.35)

Abbreviations: OR: Odds Ratio; CI: Confidence Interval; PS: Propensity Score; ADHD, Attention-deficit/hyperactivity disorder; ASD, Autism spectrum disorder; OCD, Obsessive-compulsive disorder; PTSD, Post-traumatic stress disorder.

a. Models adjusted for current age (continuous), annual income, employment, enrollment year, US census division, and race/ethnicity

b. Models adjusted for current age (continuous), annual income, employment, enrollment year, and US census division

© 2025 Lu JA et al. *JAMA Network Open*.

eTable 6. Odds Ratios of Mental Health Conditions Between Gender Diverse People Assigned Female at Birth of Any Sexual Orientation and Cisgender Heterosexual Men in the All of Us Research Program (2017-2022)

Mental Health Conditions	Unadjusted OR (95% CI)	Regression adjusted [a] OR (95% CI)	Regression adjusted [b] OR (95% CI)	PS weighted [a] OR (95% CI)
ADHD	5.14 (4.06, 6.50)	1.62 (1.26, 2.07)	2.17 (1.70, 2.76)	1.78 (1.38, 2.28)
Anxiety	3.00 (2.61, 3.45)	2.31 (2.01, 2.66)	2.81 (2.45, 3.24)	2.56 (2.22, 2.94)
ASD	12.40 (7.77, 19.79)	2.57 (1.53, 4.31)	3.72 (2.24, 6.19)	2.51 (1.42, 4.42)
Bipolar disorder	3.16 (2.52, 3.95)	2.26 (1.80, 2.85)	2.62 (2.09, 3.28)	2.87 (2.29, 3.61)
Depression	2.79 (2.43, 3.21)	2.59 (2.24, 2.98)	3.01 (2.62, 3.46)	2.86 (2.48, 3.30)
Eating disorder	7.34 (4.40, 12.24)	7.20 (4.19, 12.37)	7.94 (4.67, 13.50)	7.84 (4.40, 13.99)
OCD	6.05 (3.69, 9.91)	2.85 (1.69, 4.80)	4.29 (2.56, 7.18)	3.03 (1.74, 5.28)
Personality disorder	4.14 (3.02, 5.68)	3.35 (2.41, 4.64)	3.92 (2.84, 5.41)	4.32 (3.10, 6.02)
PTSD	4.17 (3.41, 5.09)	3.67 (2.99, 4.50)	3.81 (3.11, 4.67)	4.35 (3.55, 5.33)
Schizophrenia	0.78 (0.42, 1.47)	0.85 (0.45, 1.59)	0.70 (0.37, 1.31)	1.05 (0.56, 1.95)

Abbreviations: OR: Odds Ratio; CI: Confidence Interval; PS: Propensity Score; ADHD, Attention-deficit/hyperactivity disorder; ASD, Autism spectrum disorder; OCD, Obsessive-compulsive disorder; PTSD, Post-traumatic stress disorder.

a. Models adjusted for current age (continuous), annual income, employment, enrollment year, US census division, and race/ethnicity

b. Models adjusted for current age (continuous), annual income, employment, enrollment year, and US census division

© 2025 Lu JA et al. *JAMA Network Open*.

eTable 7. Odds Ratios of Mental Health Conditions Between Gender Diverse People Assigned Female at Birth of Any Sexual Orientation and Cisgender Heterosexual Women in the All of Us Research Program (2017-2022)

**Mental Health Conditions**	**Unadjusted OR (95% CI)**	**Regression adjusted [a] OR (95% CI)**	**Regression adjusted [b] OR (95% CI)**	**PS weighted [a] OR (95% CI)**
ADHD	5.62 (4.45, 7.10)	2.14 (1.68, 2.72)	3.01 (2.37, 3.81)	2.33 (1.83, 2.97)
Anxiety	1.79 (1.56, 2.06)	1.42 (1.24, 1.63)	1.71 (1.49, 1.96)	1.52 (1.33, 1.74)
ASD	45.90 (27.93, 75.44)	11.20 (6.14, 20.43)	16.66 (9.44, 29.39)	9.80 (5.42, 17.72)
Bipolar disorder	3.00 (2.40, 3.75)	2.02 (1.61, 2.53)	2.40 (1.92, 2.99)	2.44 (1.96, 3.05)
Depression	1.74 (1.52, 2.01)	1.68 (1.46, 1.93)	1.89 (1.64, 2.16)	1.82 (1.59, 2.09)
Eating disorder	2.25 (1.37, 3.71)	1.32 (0.79, 2.20)	1.60 (0.97, 2.65)	1.34 (0.80, 2.25)
OCD	4.80 (2.95, 7.82)	2.43 (1.48, 4.00)	3.37 (2.06, 5.52)	2.85 (1.71, 4.74)
Personality disorder	4.21 (3.08, 5.76)	2.54 (1.84, 3.51)	3.33 (2.42, 4.57)	2.85 (2.06, 3.95)
PTSD	4.51 (3.70, 5.51)	2.77 (2.26, 3.40)	3.10 (2.53, 3.79)	3.30 (2.70, 4.03)
Schizophrenia	1.89 (1.01, 3.54)	2.45 (1.31, 4.58)	1.92 (1.02, 3.59)	2.76 (1.47, 5.20)

Abbreviations: OR: Odds Ratio; CI: Confidence Interval; PS: Propensity Score; ADHD, Attention-deficit/hyperactivity disorder; ASD, Autism spectrum disorder; OCD, Obsessive-compulsive disorder; PTSD, Post-traumatic stress disorder.

a. Models adjusted for current age (continuous), annual income, employment, enrollment year, US census division, and race/ethnicity

b. Models adjusted for current age (continuous), annual income, employment, enrollment year, and US census division

© 2025 Lu JA et al. *JAMA Network Open*.

eTable 8. Odds Ratios of Mental Health Conditions Between Gender Diverse People Assigned Male at Birth of Any Sexual Orientation and Cisgender Heterosexual Men in the All of Us Research Program (2017-2022)

**Mental Health Conditions**	**Unadjusted OR (95% CI)**	**Regression adjusted [a] OR (95% CI)**	**Regression adjusted [b] OR (95% CI)**	**PS weighted [a] OR (95% CI)**
ADHD	4.29 (2.91, 6.34)	1.65 (1.11, 2.45)	2.11 (1.42, 3.13)	1.82 (1.23, 2.70)
Anxiety	2.12 (1.69, 2.66)	1.68 (1.34, 2.10)	1.95 (1.56, 2.44)	1.84 (1.47, 2.29)
ASD	8.95 (3.93, 20.38)	2.37 (1.02, 5.51)	3.24 (1.38, 7.61)	2.25 (0.92, 5.50)
Bipolar disorder	3.26 (2.32, 4.60)	2.35 (1.66, 3.33)	2.62 (1.86, 3.70)	2.70 (1.92, 3.80)
Depression	1.88 (1.49, 2.38)	1.72 (1.37, 2.17)	1.93 (1.53, 2.43)	1.86 (1.48, 2.34)
Eating disorder	3.30 (1.05, 10.38)	3.23 (1.02, 10.18)	3.53 (1.12, 11.15)	3.34 (1.04, 10.76)
OCD	8.69 (4.58, 16.48)	4.75 (2.44, 9.24)	6.50 (3.34, 12.64)	4.99 (2.55, 9.76)
Personality disorder	3.80 (2.29, 6.31)	3.15 (1.87, 5.28)	3.52 (2.09, 5.90)	3.78 (2.27, 6.28)
PTSD	2.64 (1.82, 3.82)	2.21 (1.53, 3.20)	2.25 (1.56, 3.25)	2.56 (1.79, 3.67)
Schizophrenia	0.76 (0.28, 2.04)	0.72 (0.27, 1.92)	0.65 (0.24, 1.73)	0.79 (0.29, 2.11)

Abbreviations: OR: Odds Ratio; CI: Confidence Interval; PS: Propensity Score; ADHD, Attention-deficit/hyperactivity disorder; ASD, Autism spectrum disorder; OCD, Obsessive-compulsive disorder; PTSD, Post-traumatic stress disorder.

a. Models adjusted for current age (continuous), annual income, employment, enrollment year, US census division, and race/ethnicity

b. Models adjusted for current age (continuous), annual income, employment, enrollment year, and US census division

© 2025 Lu JA et al. *JAMA Network Open*.

eTable 9. Odds Ratios of Mental Health Conditions Between Gender Diverse People Assigned Male at Birth of Any Sexual Orientation and Cisgender Heterosexual Women in the All of Us Research Program (2017-2022)

**Mental Health Conditions**	**Unadjusted OR (95% CI)**	**Regression adjusted [a] OR (95% CI)**	**Regression adjusted [b] OR (95% CI)**	**PS weighted [a] OR (95% CI)**
ADHD	4.69 (3.18, 6.92)	2.19 (1.48, 3.23)	2.92 (1.98, 4.32)	2.33 (1.57, 3.44)
Anxiety	1.26 (1.01, 1.59)	1.06 (0.85, 1.33)	1.22 (0.97, 1.52)	1.11 (0.89, 1.39)
ASD	33.14 (14.32, 76.71)	9.81 (4.05, 23.77)	14.28 (5.90, 34.56)	9.01 (3.71, 21.92)
Bipolar disorder	3.10 (2.20, 4.36)	2.10 (1.48, 2.96)	2.40 (1.71, 3.38)	2.35 (1.67, 3.30)
Depression	1.18 (0.93, 1.49)	1.14 (0.90, 1.43)	1.24 (0.98, 1.56)	1.20 (0.96, 1.51)
Eating disorder	1.01 (0.32, 3.17)	0.65 (0.21, 2.06)	0.77 (0.24, 2.42)	0.67 (0.21, 2.11)
OCD	6.90 (3.66, 13.02)	3.94 (2.06, 7.55)	5.16 (2.70, 9.86)	4.43 (2.32, 8.49)
Personality disorder	3.87 (2.34, 6.41)	2.56 (1.52, 4.32)	3.11 (1.85, 5.23)	2.66 (1.58, 4.46)
PTSD	2.86 (1.97, 4.14)	1.84 (1.25, 2.71)	2.00 (1.36, 2.95)	2.05 (1.41, 2.99)
Schizophrenia	1.83 (0.68, 4.92)	1.90 (0.71, 5.14)	1.70 (0.63, 4.55)	2.00 (0.74, 5.42)

Abbreviations: OR: Odds Ratio; CI: Confidence Interval; PS: Propensity Score; ADHD, Attention-deficit/hyperactivity disorder; ASD, Autism spectrum disorder; OCD, Obsessive-compulsive disorder; PTSD, Post-traumatic stress disorder.

a. Models adjusted for current age (continuous), annual income, employment, enrollment year, US census division, and race/ethnicity

b. Models adjusted for current age (continuous), annual income, employment, enrollment year, and US census division

© 2025 Lu JA et al. *JAMA Network Open*.

eTable 10. Odds Ratios of Mental Health Conditions Between Transgender Men of Any Sexual Orientation and Cisgender Heterosexual Men in the All of Us Research Program (2017-2022)

Mental Health Conditions	Unadjusted<br>OR (95% CI)	Regression adjusted [a]<br>OR (95% CI)	Regression adjusted [b]<br>OR (95% CI)	PS weighted [a]<br>OR (95% CI)
ADHD	2.82 (1.98, 4.01)	1.74 (1.21, 2.50)	1.78 (1.25, 2.54)	1.72 (1.20, 2.45)
Anxiety	2.07 (1.74, 2.47)	1.88 (1.60, 2.22)	1.90 (1.61, 2.25)	2.03 (1.72, 2.40)
ASD	5.09 (2.24, 11.53)	1.94 (0.83, 4.55)	2.07 (0.90, 4.75)	1.89 (0.81, 4.43)
Bipolar disorder	2.78 (2.11, 3.67)	1.92 (1.47, 2.52)	1.85 (1.41, 2.43)	2.12 (1.62, 2.77)
Depression	2.20 (1.85, 2.61)	2.11 (1.80, 2.49)	2.12 (1.80, 2.50)	2.27 (1.93, 2.67)
Eating disorder	4.42 (2.07, 9.41)	4.86 (2.27, 10.38)	4.96 (2.33, 10.54)	5.17 (2.40, 11.14)
OCD	3.42 (1.61, 7.28)	2.83 (1.31, 6.11)	2.85 (1.33, 6.11)	3.11 (1.45, 6.68)
Personality disorder	4.53 (3.17, 6.46)	3.85 (2.71, 5.47)	3.77 (2.65, 5.36)	4.34 (3.06, 6.15)
PTSD	2.47 (1.85, 3.30)	2.14 (1.61, 2.84)	2.09 (1.57, 2.78)	2.33 (1.76, 3.08)
Schizophrenia	1.76 (1.07, 2.90)	1.16 (0.71, 1.90)	1.09 (0.67, 1.78)	1.22 (0.75, 2.01)

Abbreviations: OR: Odds Ratio; CI: Confidence Interval; PS: Propensity Score; ADHD, Attention-deficit/hyperactivity disorder; ASD, Autism spectrum disorder; OCD, Obsessive-compulsive disorder; PTSD, Post-traumatic stress disorder.

a. Models adjusted for current age (continuous), annual income, employment, enrollment year, US census division, and race/ethnicity

b. Models adjusted for current age (continuous), annual income, employment, enrollment year, and US census division

© 2025 Lu JA et al. *JAMA Network Open*.

eTable 11. Odds Ratios of Mental Health Conditions Between Transgender Women of Any Sexual Orientation and Cisgender Heterosexual Women in the All of Us Research Program (2017-2022)

Mental Health Conditions	Unadjusted OR (95% CI)	Regression adjusted [a] OR (95% CI)	Regression adjusted [b] OR (95% CI)	PS weighted [a] OR (95% CI)
ADHD	1.69 (1.07, 2.67)	1.38 (0.87, 2.19)	1.42 (0.90, 2.24)	1.39 (0.88, 2.20)
Anxiety	1.03 (0.86, 1.23)	0.95 (0.80, 1.13)	0.97 (0.81, 1.16)	0.97 (0.81, 1.16)
ASD	18.33 (7.95, 42.27)	11.91 (4.91, 28.92)	12.16 (5.07, 29.20)	11.67 (4.95, 27.48)
Bipolar disorder	2.03 (1.49, 2.75)	1.34 (0.99, 1.82)	1.35 (1.00, 1.83)	1.38 (1.02, 1.87)
Depression	1.22 (1.03, 1.45)	1.12 (0.95, 1.33)	1.13 (0.96, 1.35)	1.15 (0.97, 1.36)
Eating disorder	0.37 (0.09, 1.51)	0.33 (0.08, 1.32)	0.34 (0.08, 1.36)	0.33 (0.08, 1.32)
OCD	1.51 (0.56, 4.04)	1.30 (0.48, 3.49)	1.32 (0.49, 3.56)	1.38 (0.52, 3.71)
Personality disorder	3.77 (2.57, 5.52)	2.71 (1.84, 3.99)	2.73 (1.85, 4.02)	2.80 (1.91, 4.11)
PTSD	2.65 (2.00, 3.53)	1.68 (1.25, 2.26)	1.70 (1.26, 2.28)	1.71 (1.27, 2.30)
Schizophrenia	5.74 (3.73, 8.82)	3.51 (2.26, 5.45)	3.57 (2.30, 5.52)	3.63 (2.35, 5.63)

Abbreviations: OR: Odds Ratio; CI: Confidence Interval; PS: Propensity Score; ADHD, Attention-deficit/hyperactivity disorder; ASD, Autism spectrum disorder; OCD, Obsessive-compulsive disorder; PTSD, Post-traumatic stress disorder.

a. Models adjusted for current age (continuous), annual income, employment, enrollment year, US census division, and race/ethnicity

b. Models adjusted for current age (continuous), annual income, employment, enrollment year, and US census division

© 2025 Lu JA et al. *JAMA Network Open*.

eTable 12. Rate Ratios of Mental Health Condition Counts Comparing SGM and Non-SGM Groups in the All of Us Research Program (2017-2022)

SGM Group	Reference Group	Adjusted [a] Rate Ratios (95% CI)
Cisgender sexual minority men	Cisgender heterosexual men	1.52 (1.45-1.59)
Cisgender sexual minority women	Cisgender heterosexual women	1.30 (1.26-1.34)
Gender diverse people assigned female at birth of any sexual orientation	Cisgender heterosexual men	1.68 (1.50-1.89)
Gender diverse people assigned male at birth of any sexual orientation	Cisgender heterosexual women	1.30 (1.18-1.44)
Gender diverse people assigned female at birth of any sexual orientation	Cisgender heterosexual men	1.53 (1.25-1.86)
Gender diverse people assigned male at birth of any sexual orientation	Cisgender heterosexual women	1.23 (1.04-1.45)
Transgender men of any sexual orientation	Cisgender heterosexual men	1.59 (1.36-1.84)
Transgender women of any sexual orientation	Cisgender heterosexual women	1.23 (1.07-1.40)

a. Models adjusted for current age (continuous), annual income, employment, enrollment year, US census division, and race/ethnicity

© 2025 Lu JA et al. *JAMA Network Open*.

eFigure 8. Rate Ratios of Mental Health Condition Counts Comparing SGM and Non-SGM Groups in the All of Us Research Program (2017-2022)

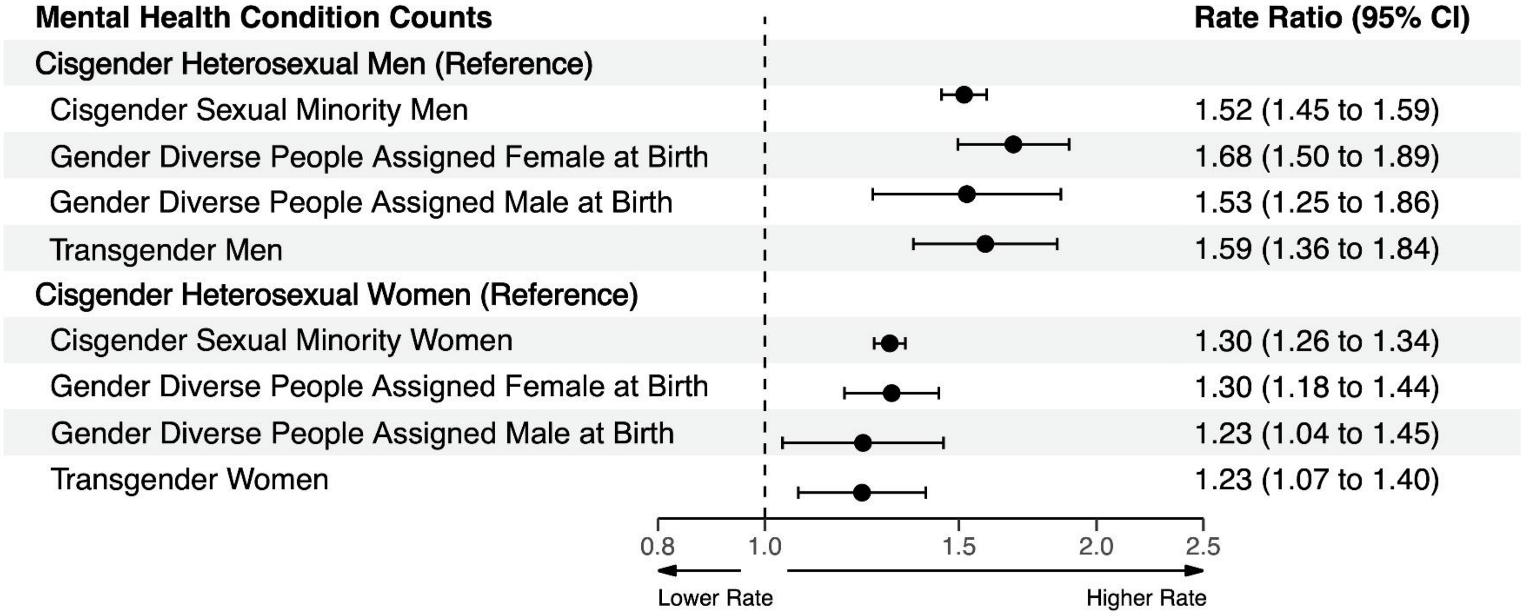

Models adjusted for current age (continuous), annual income, employment, enrollment year, US census division, and race/ethnicity

© 2025 Lu JA et al. *JAMA Network Open*.

eTable 13. Prescription Names Used with EHR Diagnosed Records to Determine Mental Health Conditions in the All of Us Research Program (2017-2022)

Mental Health Condition	FDA Approved Prescriptions[2–10]	OMOP Concept ID
Anxiety	Alprazolam	781039
	Buspirone	733301
	Clonazepam	798874
	Escitalopram	715939
	Fluoxetine	755695
	Fluvoxamine	751412
	Paroxetine	722031
	Sertraline	739138
	Venlafaxine	743670
ADHD	Atomoxetine	742185
	Clonidine	1398937
	Guanfacine	1344965
	Viloxazine	19008261
ASD	Aripiprazole	757688
	Risperidone	735979
Bipolar disorder	Aripiprazole	757688
	Asenapine	40164052
	Carbamazepine	740275
	Cariprazine	35603277
	Fluoxetine	755695
	Lamotrigine	705103
	Lithium	19124477
	Lurasidone	40230761
	Olanzapine	785788
	Quetiapine	766814
	Risperidone	735979
	Valproate	745466
	Ziprasidone	712615
Eating disorder	Fluoxetine	755695
	Lisdexamfetamine	709567
Depression	Amitriptyline	710062
	Amoxapine	713109
	Bupropion	750982
	Citalopram	797617
	Desipramine	716968
	Desvenlafaxine	717607
	Doxepin	738156
	Duloxetine	715259

© 2025 Lu JA et al. *JAMA Network Open*.

Mental Health Condition	FDA Approved Prescriptions[2–10]	OMOP Concept ID
	Escitalopram	715939
	Esketamine	1366610
	Fluoxetine	755695
	Imipramine	778268
	Isocarboxazid	781705
	Levomilnacipran	43560354
	Maprotiline	794147
	Mirtazapine	725131
	Nefazodone	714684
	Nortriptyline	721724
	Paroxetine	722031
	Phenelzine	733896
	Protriptyline	754270
	Selegiline	766209
	Sertraline	739138
	Tranylcypromine	703470
	Trazodone	703547
	Trimipramine	705755
	Venlafaxine	743670
	Vilazodone	40234834
	Vortioxetine	44507700
OCD	Clomipramine	798834
	Fluoxetine	755695
	Fluvoxamine	751412
	Paroxetine	722031
	Sertraline	739138
Personality disorder a	NA	NA
PTSD	Paroxetine	722031
	Sertraline	739138
Schizophrenia	Aripiprazole	757688
	Asenapine	40164052
	Chlorpromazine	794852
	Clozapine	800878
	Droperidol	739323
	Fluphenazine	756018
	Haloperidol	766529
	Iloperidone	19017241
	Loxapine	792263
	Olanzapine	785788
	Paliperidone	703244
	Perphenazine	733008

© 2025 Lu JA et al. *JAMA Network Open*.

Mental Health Condition	FDA Approved Prescriptions[2–10]	OMOP Concept ID
	Pimozide	745790
	Prochlorperazine	752061
	Quetiapine	766814
	Risperidone	735979
	Thioridazine	700299
	Thiothixene	700465
	Trifluoperazine	704984
	Ziprasidone	712615

Abbreviations: ADHD, Attention-deficit/hyperactivity disorder; ASD, Autism spectrum disorder; OCD, Obsessive-compulsive disorder; PTSD, Post-traumatic stress disorder; OMOP, The Observational Medical Outcomes Partnership

a. FDA did not approve medication for the treatment of personality disorder as the main condition. The results remained the same as the primary analysis under this outcome definition.

© 2025 Lu JA et al. *JAMA Network Open*.

eTable 14. Sensitivity Analysis of Mental Health Conditions Determined by Both EHR-Diagnosed Codes and Prescription Records by Sexual Orientation and Gender Identity Group in the All of Us Research Program (2017-2022)

	**Participants, No. (%)**							
Condition	Cisgender heterosexual men	Cisgender heterosexual women	Cisgender sexual minority men	Cisgender sexual minority women	Gender diverse people assigned female sex at birth of any sexual orientation	Gender diverse people assigned male sex at birth of any sexual orientation	Transgender men of any sexual orientation	Transgender women of any sexual orientation
Total (n=269 947), No. a	94 998	152 760	8075	11572	884	365	638	655
Anxiety	9074 (9.6)	24 079 (15.8)	1240 (15.4)	2425 (21.0)	225 (25.5)	64 (17.5)	119 (18.7)	91 (13.9)
ADHD	414 (0.4)	553 (0.4)	60 (0.7)	148 (1.3)	22 (2.5)	< 20 [a]	< 20 [a]	< 20 [a]
ASD	62 (0.1)	25 (<0.01)	< 20 [a]	22 (0.2)	< 20 [a]	< 20 [a]	< 20 [a]	< 20 [a]
Bipolar disorder	2344 (2.5)	3945 (2.6)	425 (5.3)	814 (7.0)	70 (7.9)	28 (7.7)	45 (7.1)	35 (5.3)
Eating disorder	45 (<0.01)	367 (0.2)	< 20 [a]	76 (0.7)	< 20 [a]	< 20 [a]	< 20 [a]	< 20 [a]
Depression	11 863 (12.5)	28 428 (18.6)	1690 (20.9)	2817 (24.3)	248 (28.1)	74 (20.3)	147 (23.0)	123 (18.8)
OCD	202 (0.2)	414 (0.3)	29 (0.4)	70 (0.6)	< 20 [a]	< 20 [a]	< 20 [a]	< 20 [a]
Personality disorder	1131 (1.2)	1789 (1.2)	168 (2.1)	360 (3.1)	42 (4.8)	< 20 [a]	33 (5.2)	28 (4.3)
PTSD	1199 (1.3)	1889 (1.2)	122 (1.5)	348 (3.0)	39 (4.4)	< 20 [a]	< 20 [a]	< 20 [a]
Schizophrenia	1112 (1.2)	749 (0.5)	119 (1.5)	124 (1.1)	< 20 [a]	< 20 [a]	< 20 [a]	< 20 [a]

Abbreviations: EHR, electronic health record; ADHD, Attention-deficit/hyperactivity disorder; ASD, Autism spectrum disorder; OCD, Obsessive-compulsive disorder; PTSD, Post-traumatic stress disorder.

a. Groups with 1 to 19 participants were expressed as having fewer than 20 in accordance with *All of Us* policy.

© 2025 Lu JA et al. *JAMA Network Open*.

eTable 15. Sensitivity Analysis of Mental Health Conditions Determined by Both EHR-Diagnosed Codes and Prescription Records: Odds Ratios for Mental Health Conditions Comparing SGM and Non-SGM Groups in the All of Us Research Program (2017-2022)

	**Regression adjusted [a] Odds Ratios (95% CI)**									
Comparison groups	ADHD	Anxiety	ASD	Bipolar disorder	Depression	Eating disorder	OCD	Personality disorder	PTSD	Schizophrenia
Cisgender sexual minority men VS Cisgender heterosexual men	1.22 (0.92, 1.60)	1.60 (1.50, 1.70)	1.03 (0.51, 2.07)	1.92 (1.73, 2.14)	1.80 (1.70, 1.90)	2.11 (1.04, 4.31)	1.37 (0.91, 2.05)	1.69 (1.43, 2.00)	1.21 (1.00, 1.46)	1.15 (0.95, 1.40)
Cisgender heterosexual women VS Cisgender heterosexual women	1.91 (1.58, 2.32)	1.25 (1.19, 1.31)	5.44 (2.96, 9.99)	2.07 (1.91, 2.25)	1.38 (1.33, 1.45)	1.85 (1.42, 2.40)	1.51 (1.16, 1.96)	1.96 (1.73, 2.21)	1.67 (1.48, 1.89)	1.90 (1.55, 2.31)
Gender diverse people assigned female at birth of any sexual orientation VS Cisgender heterosexual men	1.60 (1.00, 2.54)	2.40 (2.05, 2.80)	4.10 (1.98, 8.48)	2.41 (1.87, 3.11)	2.50 (2.16, 2.90)	2.58 (0.63, 10.51)	2.79 (1.50, 5.22)	3.35 (2.41, 4.64)	3.14 (2.24, 4.41)	0.90 (0.47, 1.75)
Gender diverse people assigned female at birth of any sexual orientation VS Cisgender heterosexual women	2.44 (1.55, 3.84)	1.40 (1.20, 1.63)	27.01 (11.23, 64.94)	2.06 (1.60, 2.64)	1.61 (1.39, 1.86)	0.43 (0.11, 1.75)	2.44 (1.35, 4.41)	2.54 (1.84, 3.51)	2.12 (1.52, 2.97)	2.65 (1.37, 5.14)
Gender diverse people assigned male at birth of any sexual orientation VS Cisgender heterosexual men	2.21 (1.13, 4.29)	1.57 (1.20, 2.06)	3.66 (1.10, 12.19)	2.37 (1.60, 3.51)	1.63 (1.26, 2.10)	3.18 (0.43, 23.57)	3.27 (1.30, 8.25)	3.15 (1.87, 5.28)	1.99 (1.08, 3.67)	0.42 (0.10, 1.73)
Gender diverse people assigned male at birth of any sexual orientation VS Cisgender heterosexual women	3.27 (1.69, 6.33)	0.95 (0.72, 1.24)	20.85 (6.03, 72.03)	2.06 (1.39, 3.07)	1.07 (0.83, 1.38)	0.63 (0.09, 4.56)	2.85 (1.15, 7.02)	2.56 (1.52, 4.32)	1.61 (0.86, 3.02)	1.14 (0.28, 4.65)
Transgender men of any sexual orientation VS Cisgender heterosexual men	1.53 (0.74, 3.15)	2.07 (1.70, 2.51)	2.85 (0.84, 9.64)	2.14 (1.58, 2.89)	2.10 (1.76, 2.51)	7.82 (2.39, 25.65)	2.43 (0.88, 6.68)	3.85 (2.71, 5.47)	1.31 (0.72, 2.38)	1.34 (0.81, 2.22)
Transgender women of any sexual orientation VS Cisgender heterosexual women	1.51 (0.62, 3.68)	0.84 (0.68, 1.05)	6.22 (0.82, 47.35)	1.48 (1.05, 2.08)	0.97 (0.80, 1.18)	0.57 (0.08, 4.10)	1.48 (0.47, 4.63)	2.71 (1.84, 3.99)	1.06 (0.60, 1.87)	3.80 (2.37, 6.11)

a. Models adjusted for current age (continuous), annual income, employment, enrollment year, US census division, and race/ethnicity

© 2025 Lu JA et al. *JAMA Network Open*.

eTable 16. Sensitivity Analysis of Mental Health Conditions Determined by Both EHR-Diagnosed Codes and Prescription Records: Rate Ratios for Mental Health Condition Counts Comparing SGM and Non-SGM Groups in the All of Us Research Program (2017-2022)

**SGM Group**	**Reference Group**	**Adjusted [a] Rate Ratios (95% CI)**
Cisgender sexual minority men	Cisgender heterosexual men	1.53 (1.45-1.62)
Cisgender sexual minority women	Cisgender heterosexual women	1.33 (1.28-1.38)
Gender diverse people assigned female at birth of any sexual orientation	Cisgender heterosexual men	1.76 (1.53-2.03)
Gender diverse people assigned male at birth of any sexual orientation	Cisgender heterosexual women	1.31 (1.16-1.47)
Gender diverse people assigned female at birth of any sexual orientation	Cisgender heterosexual men	1.58 (1.24-2.00)
Gender diverse people assigned male at birth of any sexual orientation	Cisgender heterosexual women	1.24 (1.02-1.52)
Transgender men of any sexual orientation	Cisgender heterosexual men	1.72 (1.43-2.06)
Transgender women of any sexual orientation	Cisgender heterosexual women	1.18 (1.00-1.38)

a. Models adjusted for current age (continuous), annual income, employment, enrollment year, US census division, and race/ethnicity

© 2025 Lu JA et al. *JAMA Network Open*.

eFigure 9. Sensitivity Analysis of Mental Health Conditions Determined by Both EHR Diagnosed Codes and Prescription Records: Risk Ratios for Mental Health Condition Counts Comparing SGM and Non-SGM Groups in the All of Us Research Program (2017-2022)

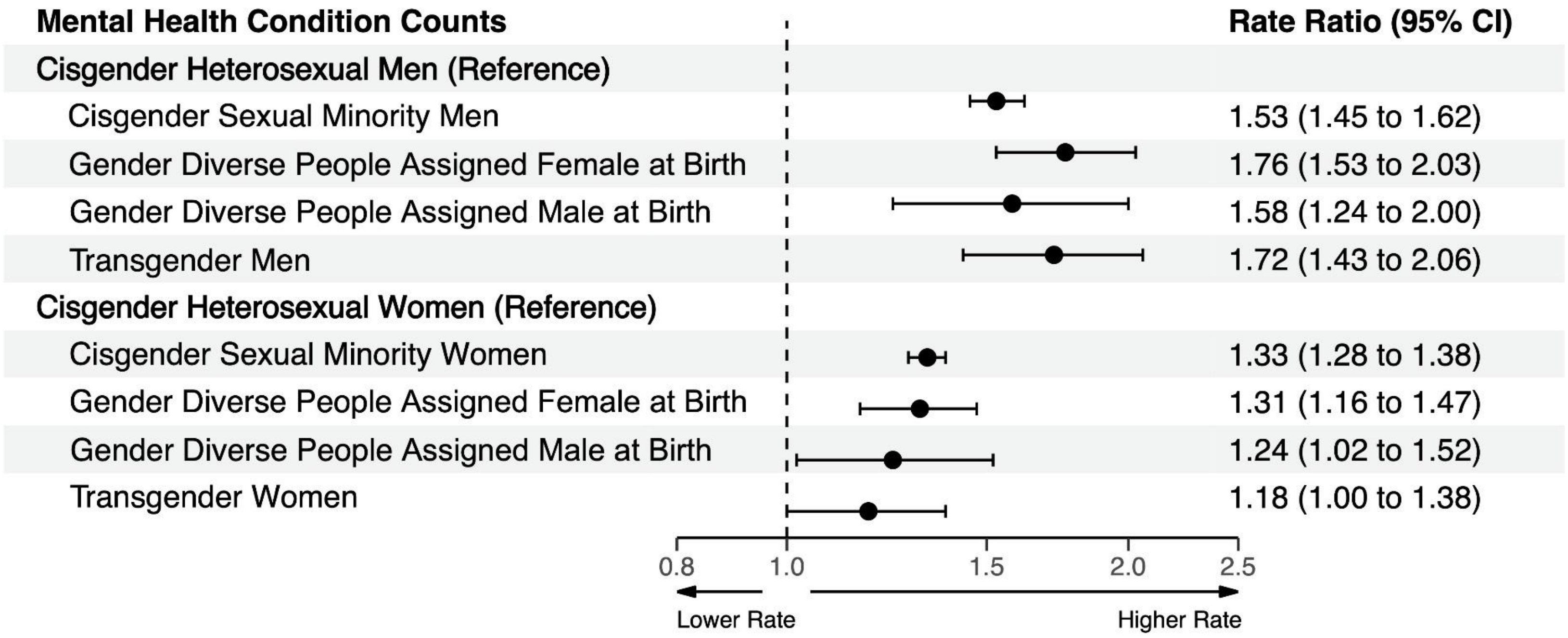

Models adjusted for current age (continuous), annual income, employment, enrollment year, US census division, and race/ethnicity

© 2025 Lu JA et al. *JAMA Network Open*.

eTable 17. EHR Visit Types and Data Generalizations for Classifying Inpatient and Outpatient Visits in the All of Us Research Program (2017-2022)

**Binary Visit Type**	**Detailed Visit Type**
Inpatient Categories	Ambulatory Oncological Radiation Clinic / Center
	Ambulatory Recovery Care Clinic / Center
	Emergency Room and Inpatient Visit
	Hospital
	Inpatient Hospital
	Inpatient Visit
	Intensive Care
	Non-hospital institution Visit
	Nursing Facility
Outpatient Categories	Ambulatory Clinic / Center
	Ambulatory Endoscopy Clinic / Center
	Ambulatory Infusion Therapy Clinic / Center
	Ambulatory Magnetic Resonance Imaging (MRI) Clinic / Center
	Ambulatory Mammography Clinic / Center
	Ambulatory Occupational Medicine Clinic / Center
	Ambulatory Oncology Clinic / Center
	Ambulatory Pain Clinic / Center
	Ambulatory Physical Therapy Clinic / Center
	Ambulatory Radiology Clinic / Center
	Ambulatory Rehabilitation Visit
	Ambulatory Surgical Center
	Case Management Visit
	Emergency Room - Hospital
	Emergency Room Visit
	Home Visit
	Laboratory Visit
	Observation Room
	Office Visit
	Outpatient Hospital
	Outpatient Visit
	Pharmacy Visit
	Telehealth
Unknown	Unknown Value (but present in data)

© 2025 Lu JA et al. *JAMA Network Open*.

eTable 18. Sensitivity Analysis of Mental Health Conditions Determined by Both EHR Diagnosed Codes and Inpatient and Outpatient Records by Sexual Orientation and Gender Identity Group in the All of Us Research Program (2017-2022)

	Participants, No. (%)							
Condition	Cisgender heterosexual men	Cisgender heterosexual women	Cisgender sexual minority men	Cisgender sexual minority women	Gender diverse people assigned female sex at birth of any sexual orientation	Gender diverse people assigned male sex at birth of any sexual orientation	Transgender men of any sexual orientation	Transgender women of any sexual orientation
Total (n=269 947), No. a	94 998	152 760	8075	11572	884	365	638	655
Anxiety	3733 (3.9)	8384 (5.5)	467 (5.8)	749 (6.5)	61 (6.9)	< 20	45 (7.1)	32 (4.9)
ADHD	306 (0.3)	438 (0.3)	50 (0.6)	101 (0.9)	< 20	< 20	< 20	< 20
ASD	42 (<0.01)	< 20	< 20	< 20	< 20	< 20	< 20	< 20
Bipolar disorder	969 (1.0)	1785 (1.2)	182 (2.3)	299 (2.6)	24 (2.7)	< 20	< 20	< 20
Eating disorder	< 20	121 (0.1)	< 20	27 (0.2)	< 20	< 20	< 20	< 20
Depression	4476 (4.7)	9961 (6.5)	635 (7.9)	883 (7.6)	74 (8.4)	22 (6.0)	58 (9.1)	38 (5.8)
OCD	69 (0.1)	100 (0.1)	< 20	< 20	< 20	< 20	< 20	< 20
Personality disorder	301 (0.3)	453 (0.3)	40 (0.5)	101 (0.9)	< 20	< 20	< 20	< 20
PTSD	814 (0.9)	955 (0.6)	88 (1.1)	195 (1.7)	28 (3.2)	< 20	< 20	< 20
Schizophrenia	445 (0.5)	298 (0.2)	48 (0.6)	40 (0.3)	< 20	< 20	< 20	< 20

Abbreviations: ADHD, Attention-deficit/hyperactivity disorder; ASD, Autism spectrum disorder; OCD, Obsessive-compulsive disorder; PTSD, Post-traumatic stress disorder.

a. Groups with 1 to 19 participants were expressed as having fewer than 20 in accordance with All of Us policy.

© 2025 Lu JA et al. *JAMA Network Open*.

eTable 19. Sensitivity Analysis of Mental Health Conditions Determined by Both EHR Diagnosed Codes and Inpatient and Outpatient Records: Odds Ratios for Mental Health Conditions Comparing SGM and Non-SGM Groups in the All of Us Research Program (2017-2022)

	Regression adjusted [a] OR (95% CI)									
Comparison groups	ADHD	Anxiety	ASD	Bipolar disorder	Depres sion	Eating disorder	OCD	Personalit y disorder	PTSD	Schizophrenia
Cisgender sexual minority men VS Cisgender heterosexual men	1.57 (1.16, 2.11)	1.43 (1.30, 1.58)	1.95 (0.98, 3.90)	1.95 (1.65, 2.30)	1.70 (1.56, 1.85)	2.25 (0.63, 8.03)	1.57 (0.81, 3.05)	1.44 (1.03, 2.02)	1.30 (1.04, 1.62)	1.12 (0.83, 1.52)
Cisgender heterosexual women VS Cisgender heterosexual women	1.87 (1.48, 2.36)	1.16 (1.07, 1.26)	10.35 (3.52, 30.45)	1.77 (1.55, 2.02)	1.28 (1.19, 1.38)	1.60 (1.03, 2.51)	1.13 (0.60, 2.11)	1.89 (1.50, 2.38)	1.82 (1.54, 2.15)	1.72 (1.22, 2.42)
Gender diverse people assigned female at birth of any sexual orientation VS Cisgender heterosexual men	1.38 (0.68, 2.78)	1.66 (1.28, 2.17)	2.90 (1.11, 7.53)	1.99 (1.32, 3.02)	2.03 (1.60, 2.59)	12.86 (2.42, 68.36)	0.90 (0.12, 6.65)	3.06 (1.65, 5.68)	3.34 (2.23, 5.01)	1.16 (0.42, 3.19)
Gender diverse people assigned female at birth of any sexual orientation VS Cisgender heterosexual women	1.41 (0.71, 2.80)	1.13 (0.87, 1.47)	16.32 (4.46, 59.74)	1.62 (1.08, 2.44)	1.42 (1.12, 1.80)	0.90 (0.22, 3.75)	0.83 (0.11, 6.09)	2.16 (1.19, 3.94)	2.68 (1.79, 3.99)	3.57 (1.29, 9.88)
Gender diverse people assigned male at birth of any sexual orientation VS Cisgender heterosexual men	2.61 (1.16, 5.91)	1.10 (0.67, 1.79)	1.79 (0.23, 13.63)	2.09 (1.11, 3.93)	1.39 (0.90, 2.14)	NA [b]	2.57 (0.33, 19.85)	1.94 (0.60, 6.21)	1.05 (0.38, 2.89)	0.63 (0.09, 4.58)
Gender diverse people assigned male at birth of any sexual orientation VS Cisgender heterosexual women	2.82 (1.25, 6.37)	0.78 (0.48, 1.27)	14.32 (1.80, 113.71)	1.73 (0.92, 3.24)	1.00 (0.65, 1.55)	NA [b]	2.41 (0.32, 18.18)	1.52 (0.47, 4.87)	1.02 (0.37, 2.80)	1.69 (0.23, 12.37)
Transgender men of any sexual orientation VS Cisgender heterosexual men	2.22 (0.97, 5.10)	1.93 (1.42, 2.61)	7.50 (2.82, 19.96)	1.48 (0.83, 2.66)	2.18 (1.67, 2.85)	8.60 (1.21, 61.00)	1.83 (0.24, 13.70)	3.26 (1.60, 6.62)	2.56 (1.50, 4.36)	0.98 (0.36, 2.62)
Transgender women of any sexual orientation VS Cisgender heterosexual women	2.02 (0.83, 4.93)	0.77 (0.54, 1.11)	NA [b]	1.18 (0.68, 2.07)	0.80 (0.58, 1.11)	1.38 (0.18, 10.37)	NA [b]	3.50 (1.83, 6.70)	2.35 (1.37, 4.02)	1.47 (0.47, 4.65)

a. Models adjusted for current age (continuous), annual income, employment, enrollment year, US census division, and race/ethnicity
b. Models did not converge.

Abbreviations: ADHD, Attention-deficit/hyperactivity disorder; ASD, Autism spectrum disorder; OCD, Obsessive-compulsive disorder; PTSD, Post-traumatic stress disorder.

© 2025 Lu JA et al. *JAMA Network Open*.

eTable 20. Sensitivity Analysis of Mental Health Conditions Determined by Both EHR-Diagnosed Codes and Inpatient and Outpatient Records: Rate Ratios for Mental Health Condition Counts Comparing SGM and Non-SGM Groups in the All of Us Research Program (2017-2022)

SGM Group	Reference Group	Adjusted [a] Rate Ratios (95% CI)
Cisgender sexual minority men	Cisgender heterosexual men	1.48 (1.35-1.62)
Cisgender sexual minority women	Cisgender heterosexual women	1.31 (1.23-1.41)
Gender diverse people assigned female at birth of any sexual orientation	Cisgender heterosexual men	1.63 (1.29-2.05)
Gender diverse people assigned male at birth of any sexual orientation	Cisgender heterosexual women	1.25 (1.02-1.53)
Gender diverse people assigned female at birth of any sexual orientation	Cisgender heterosexual men	1.30 (0.87-1.94)
Gender diverse people assigned male at birth of any sexual orientation	Cisgender heterosexual women	1.06 (0.74-1.52)
Transgender men of any sexual orientation	Cisgender heterosexual men	2.09 (1.58-2.76)
Transgender women of any sexual orientation	Cisgender heterosexual women	1.28 (0.99-1.67)

a. Models adjusted for current age (continuous), annual income, employment, enrollment year, US census division, and race/ethnicity

© 2025 Lu JA et al. *JAMA Network Open*.

eFigure 10. Sensitivity Analysis of Mental Health Conditions Determined by Both EHR Diagnosed Codes and Inpatient and Outpatient Records: Risk Ratios for Mental Health Condition Counts Comparing SGM and Non-SGM Groups in the All of Us Research Program (2017-2022)

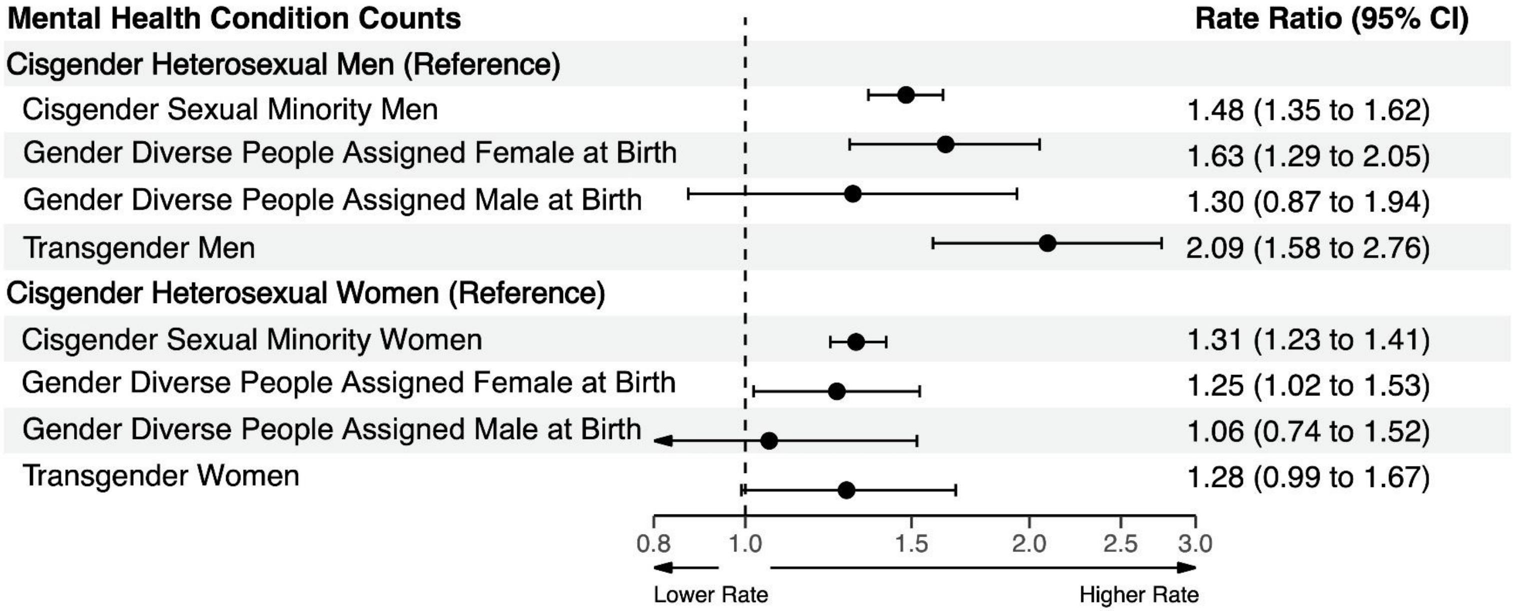

Models adjusted for current age (continuous), annual income, employment, enrollment year, US census division, and race/ethnicity

© 2025 Lu JA et al. *JAMA Network Open*.

eFigure 11. Sensitivity Analysis on Adjusted Odds Ratios of Mental Health Conditions Among Sexual and Gender Minority Participants in the All of Us Research Program (2017-2022): Part 1

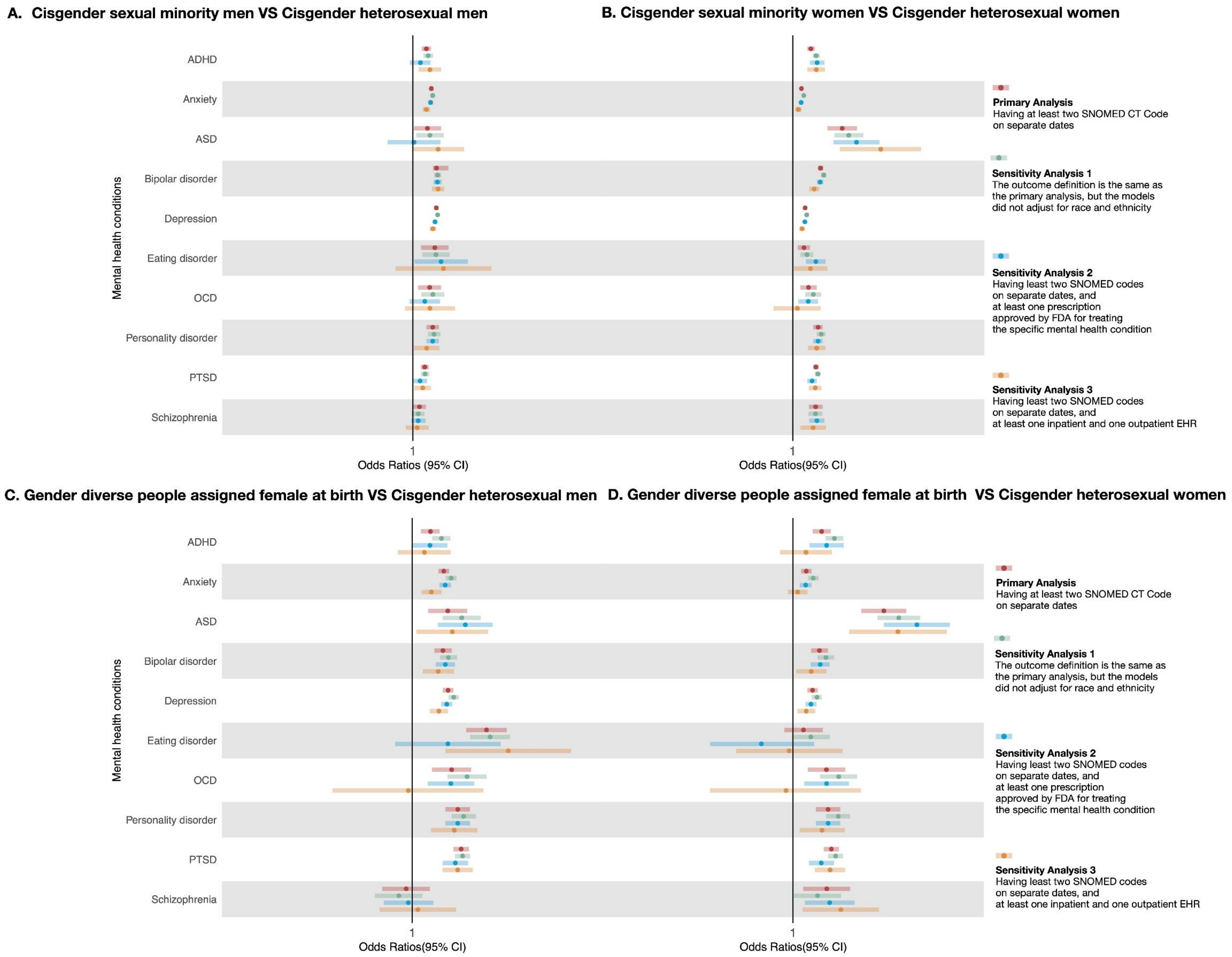

Abbreviations: ADHD, Attention-deficit/hyperactivity disorder; ASD, Autism spectrum disorder; OCD, Obsessive-compulsive disorder; PTSD, Post-traumatic stress disorder.

© 2025 Lu JA et al. *JAMA Network Open*.

eFigure 12. Sensitivity Analysis on Adjusted Odds Ratios of Mental Health Conditions Among Sexual and Gender Minority Participants in the All of Us Research Program (2017-2022): Part 2

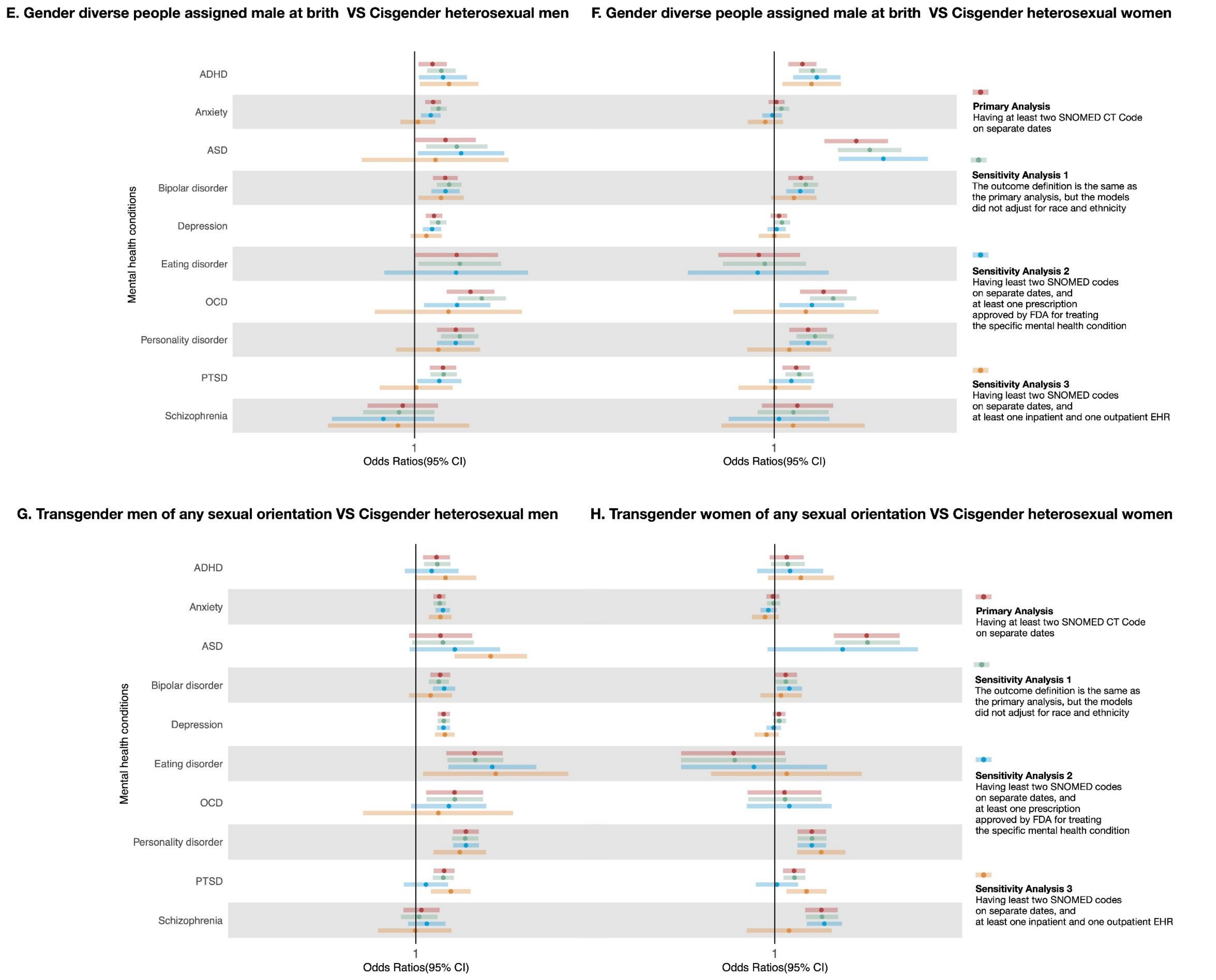

Abbreviations: ADHD, Attention-deficit/hyperactivity disorder; ASD, Autism spectrum disorder; OCD, Obsessive-compulsive disorder; PTSD, Post-traumatic stress disorder.

Estimates for sensitivity analysis 3 are missing for eating disorder in panel E; ASD and eating disorder in panel F; and ASD and OCD in panel H because the models did not converge.

© 2025 Lu JA et al. *JAMA Network Open*.

eTable 21. Characteristics of Intersex Participants With and Without Electronic Health Records the All of Us Research Program (2017-2022)

	Participants, No. (%) [j]		
**Characteristic**	**Overall**	**With Electronic Health Record**	**Without Electronic Health Record**
**Total No. of participants**	92	57	35
**Age, median (IQR), year**	50 (38, 63)	50 (38, 62)	49 (38, 65)
**Race and ethnicity [a, b, c]**			
African American or Black	23 (25.0)	< 20	< 20
Asian	< 20	< 20	< 20
Hispanic or Latinx	< 20	< 20	< 20
Middle Eastern or North African	< 20	< 20	< 20
Native Hawaiian or Other Pacific Islander	< 20	< 20	< 20
White	40 (43.5)	32 (56.1)	< 20
**Sexual orientation [a, b]**			
Asexual	< 20	< 20	< 20
Bisexual	< 20	< 20	< 20
Gay	< 20	< 20	< 20
Lesbian	< 20	< 20	< 20
Mostly straight	< 20	< 20	< 20
Queer	< 20	< 20	< 20
Polysexual, omnisexual, sapiosexual, or pansexual	< 20	< 20	< 20
Straight	37 (40.2)	< 20	< 20
Two-spirit	< 20	< 20	< 20
**Gender identity [a]**			
Genderfluid, genderqueer, gender variant, unsure, specific gender, or two-spirit	< 20	< 20	< 20
Man	21 (22.8)	< 20	< 20
Nonbinary	21 (22.8)	< 20	< 20
Transgender	21 (22.8)	< 20	< 20
Woman	24 (26.1)	< 20	< 20
**Annual household income, $ [d]**			
<25 000	40 (43.5)	32 (56.1)	< 20
25 000-49 999	< 20	< 20	< 20
50 000-99 999	< 20	< 20	< 20
100 000-149 999	< 20	< 20	< 20
>150000	< 20	< 20	< 20
Prefer to not answer or skipped	< 20	< 20	< 20
**Some college or higher [e]**	48 (52.2)	31 (54.4)	< 20
**Employed for wages [f]**	< 20	< 20	< 20
**Own a home [g]**	20 (21.7)	< 20	< 20
**Health insurance [h]**	68 (73.9)	49 (86.0)	< 20
**Enrollment year [i]**			
2017	< 20	< 20	< 20
2018	28 (30.4)	20 (35.1)	< 20
2019	29 (31.5)	< 20	< 20
2020	< 20	< 20	< 20
2021	< 20	< 20	< 20
2022	< 20	< 20	< 20

Abbreviations: IQR, interquartile range

© 2025 Lu JA et al. *JAMA Network Open*.

a. Categories are not mutually exclusive; they do not sum to the column total because participants may self-identify in multiple groups.

b. Groups with 1 to 19 participants were described as having fewer than 20 in accordance with *All of Us* policy.

c. Race and ethnicity were self-reported by the item, “Which categories describe you?” Possible answers included: African American or Black, Asian, Hispanic or Latinx, Middle Eastern or North African, Native Hawaiian or Other Pacific Islander, White. Participants could select multiple categories and were assigned to each selected racial or ethnic group, resulting in non-mutually exclusive classifications.

d. Annual household income was measured by the item, “What is your annual household income from all sources?” Possible answers included: less than 10k, 10k-25k, 25k-35k, 35k-50k, 50k-75k, 75k-100k, 100k-150k, 150k-200k, more than 200k, Skip, Prefer Not To Answer.

e. Education level was measured by the item, “What is the highest grade or year of school you completed?” Possible answers included: Never Attended, One Through Four, Five Through Eight, Nine Through Eleven, Twelve Or GED, College One to Three, College Graduate, Advanced Degree, Skip, Prefer Not To Answer. Participants answered, “College One to Three” and above were classified as “Some college or higher.”

f. Employment status was measured by the item, “What is your current employment status?” Possible answers included: Employed For Wages, Retired, Unable To Work, Self Employed, Out Of Work One Or More, Student, Homemaker, Out Of Work Less Than One, Skip, Prefer Not To Answer.

g. House ownership was measured by the item, “Do you own or rent the place where you live?” Possible answers included: Own, Rent, Other Arrangement, Don't Know, Skip, Prefer Not To Answer.

h. Insurance status was measured by the item, “Are you covered by health insurance or some other kind of health care plan?” Possible answers included: Yes, No, Skip, Prefer Not To Answer.

i. 2017 and 2022 were combined with 2018 and 2021, respectively, to ensure sufficient statistical power for regression analysis.

j. Sexual and gender minority (SGM) and non-SGM groups were defined based on sex assigned at birth, sexual orientation, and gender identity. Cisgender sexual minority participants included those who did not select “straight” as their only sexual orientation and chose other sexual orientation options. Detailed definitions are provided in eTable 2.

© 2025 Lu JA et al. *JAMA Network Open*.

eTable 22. Electronic Health Record Diagnosed Mental Health Intersex participants with any sexual orientation in All of Us Research Program (2017-2022)

	**Participants, No. (%)**
**Condition**	**Intersex participants with any sexual orientation**
**Total (n=57), No. [a]**	57
**Anxiety**	21 (36.8)
**ADHD**	< 20
**ASD**	< 20
**Bipolar disorder**	< 20
**Eating disorder**	< 20
**Depression**	20 (35.1)
**OCD**	< 20
**Personality disorder**	< 20
**PTSD**	< 20
**Schizophrenia**	< 20

Abbreviations: ADHD, attention-deficit/hyperactivity disorder; ASD, autism spectrum disorder; OCD, obsessive-compulsive disorder; PTSD, post-traumatic stress disorder.

a. Groups with 1 to 19 participants were described as having fewer than 20 in accordance with *All of Us* policy.

© 2025 Lu JA et al. *JAMA Network Open*.

eReferences.

1. Andrade C. Mean Difference, Standardized Mean Difference (SMD), and Their Use in Meta-Analysis: As Simple as It Gets. *J Clin Psychiatry*. 2020;81(5):11349. doi:10.4088/JCP.20f13681

2. The Food and Drug Administration (FDA). Depression Medicines. October 30, 2023. Accessed February 27, 2024. https://www.fda.gov/consumers/womens-health-topics/depression-medicines

3. Anxiety and Depression Association of America. FDA approved prescriptions for anxiety disorders. Accessed February 27, 2024. https://adaa.org/sites/default/files/Medications-Chart_updated-1209.pdf

4. Christian R, Saavedra L, Gaynes BN, et al. *Future Research Needs for First- and Second-Generation Antipsychotics for Children and Young Adults*. Agency for Healthcare Research and Quality (US); 2012. Accessed February 27, 2024. http://www.ncbi.nlm.nih.gov/books/NBK84660/

5. American Psychological Association (APA). Medications for PTSD. https://www.apa.org. Accessed February 27, 2024. https://www.apa.org/ptsd-guideline/treatments/medications

6. Woody EZ, Hoffman KL, Szechtman H. Obsessive compulsive disorder (OCD): Current treatments and a framework for neurotherapeutic research. In: *Advances in Pharmacology*. Vol 86. Elsevier; 2019:237-271. doi:10.1016/bs.apha.2019.04.003

7. Bello NT, Yeomans BL. Safety of pharmacotherapy options for bulimia nervosa and binge eating disorder. *Expert Opin Drug Saf*. 2018;17(1):17-23. doi:10.1080/14740338.2018.1395854

8. Woodward S. The Voice of the Patient A series of reports from the U.S. Food and Drug Administration's Patient-Focused Drug Development Initiative Autism. https://www.fda.gov/media/111099/download

9. Treating and Dealing with ADHD. *FDA*. Published online August 23, 2023. Accessed February 27, 2024. https://www.fda.gov/consumers/consumer-updates/treating-and-dealing-adhd

10. Butler M, Urosevic S, Desai P, et al. *Treatment for Bipolar Disorder in Adults: A Systematic Review*. Agency for Healthcare Research and Quality (US); 2018. Accessed February 27, 2024. http://www.ncbi.nlm.nih.gov/books/NBK532183/

© 2025 Lu JA et al. *JAMA Network Open*.
