# Supplementary material for: Mental Health Disparities by Sexual Orientation and Gender Identity in the All of Us Research Program
Source: JAMA Netw Open. 2025 Jan 29;8(1):e2456264. doi: 10.1001/jamanetworkopen.2024.56264 (PMC11780479; doi:10.1001/jamanetworkopen.2024.56264)
Supplement: Supplement 2. — Data Sharing Statement [file jamanetwopen-e2456264-s002.pdf]

Data Sharing Statement

Lu. Mental Health Disparities by Sexual Orientation and Gender Identity in the All of Us Research Program. *JAMA Netw Open*. Published January 29, 2025.
doi:10.1001/jamanetworkopen.2024.56264

Data
Data available: Yes
Data types: Deidentified participant data
How to access data: The data are publicly available to authorized users with controlled tier access through the All of Us Researcher Workbench at https://www.researchallofus.org/data-tools/workbench/
When available: With publication

Supporting Documents
Document types: Statistical/analytic code
How to access documents: The code is publicly available to authorized users with controlled tier access through the All of Us Researcher Workbench at https://www.researchallofus.org/data-tools/workbench/
When available: With publication

Additional Information
Who can access the data: The data are publicly available to authorized users with controlled tier access through the All of Us Researcher Workbench at https://www.researchallofus.org/data-tools/workbench/
Types of analyses: Data and code will be made available for replication purposes.
Mechanisms of data availability: Data and code will be made available after approval by the All of Us Research Program for authorized users with controlled tier access.
